# Siroheme synthase orients substrates for dehydrogenase and chelatase activities in a common active site

**DOI:** 10.1038/s41467-020-14722-1

**Published:** 2020-02-13

**Authors:** Joseph M. Pennington, Michael Kemp, Lauren McGarry, Yu Chen, M. Elizabeth Stroupe

**Affiliations:** 1 Department of Biological Science and Institute of Molecular Biophysics, Florida State University, 91 Chieftan Way, Tallahassee, FL 32306 USA; 2 Department of Molecular Medicine, University of South Florida College of Medicine, 12901 Bruce B. Downs Blvd, MDC 3522, Tampa, FL 33612 USA

**Keywords:** Enzyme mechanisms, X-ray crystallography

## Abstract

Siroheme is the central cofactor in a conserved class of sulfite and nitrite reductases that catalyze the six-electron reduction of sulfite to sulfide and nitrite to ammonia. In *Salmonella enterica* serovar Typhimurium, siroheme is produced by a trifunctional enzyme, siroheme synthase (CysG). A bifunctional active site that is distinct from its methyltransferase activity catalyzes the final two steps, NAD^+^-dependent dehydrogenation and iron chelation. How this active site performs such different chemistries is unknown. Here, we report the structures of CysG bound to precorrin-2, the initial substrate; sirohydrochlorin, the dehydrogenation product/chelation substrate; and a cobalt-sirohydrochlorin product. We identified binding poses for all three tetrapyrroles and tested the roles of specific amino acids in both activities to give insights into how a bifunctional active site catalyzes two different chemistries and acts as an iron-specific chelatase in the final step of siroheme synthesis.

## Abstract (web summary)

Siroheme is an essential bacterial iron tetrapyrrole used by siroheme-dependent sulfite and nitrite reductases. Here the authors shed light on the catalytic mechanisms of siroheme synthase through the structures of the bifunctional dehydrogenase/chelatase CysG module bound to its substrate, precorrin-2, the product/substrate sirohydrochlorin, and cobalt-sirohydrochlorin.

## Introduction

Siroheme is the modified isobacteriochlorin tetrapyrrole used by siroheme-dependent sulfite and nitrite reductases (SiR/NiRs) in catalyzing the six-electron reduction of sulfite to sulfide or nitrite to ammonia^1^. Dissimilatory siroheme-dependent SiRs and NiRs direct the terminal electron transfer in sulfate or nitrate-based anaerobic respiration in sulfur or nitrogen-reducing microorganisms^2^. Assimilatory siroheme-dependent SiRs and NiRs prepare sulfur and nitrogen for incorporation into biomolecules in organisms as diverse as proteobacteria and plants^2^. This iron-containing prosthetic group is also a precursor to heme or heme *d*_1_ in the alternative heme biosynthesis route used by some sulfate-reducing or denitrifying bacteria^3,4^.

The acetyl and propionyl substituents on the pyrrole rings are not decarboxylated^5^, so siroheme is more similar to the nickel-containing tetrapyrrole F430^6^ than to more familiar protoporphyrin-derived tetrapyrroles. Taken together, these observations suggest that siroheme is more evolutionarily ancient than heme, from a time before an oxygen-rich environment. In addition, siroheme is essential in the disease causative *Mycobacterium tuberculosis* because that organism depends on reduced sulfur in mycothiol as part of its defense against oxidative stresses inflicted by its host’s macrophages^7^.

**Fig. 1:**
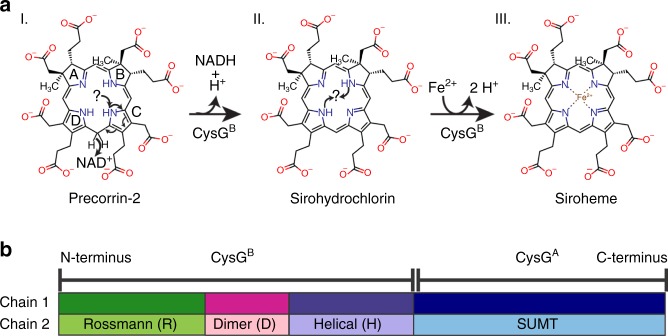
Overview of siroheme synthesis by the CysG homodimer. **a** Chemical structures and scheme for the transformation from precorrin-2 to siroheme. **b** Domain architecture of CysG. The Rossmann fold (R) is colored green throughout; the dimerization domain (d) is colored pink throughout; the all-helical domain (h) is purple throughout; and CysG^A^ is blue throughout. Chain 1 of the dimer is shaded dark throughout and chain 2 of the dimer is shaded light throughout.

Siroheme is synthesized from the common tetrapyrrole precursor uroporphyrinogen III (uro’gen III). First, a methyl group is transferred to C2, then to C7, from two molecules of *S-*adenosyl methionine (SAM) to make precorrin-2, the precursor to cobalamin and siroheme^8^. Next, a proton is removed from a pyrrole nitrogen and a hydride is abstracted from C15 via NAD^+^-dependent dehydrogenation to make sirohydrochlorin, the final precursor to siroheme (Fig. 1a). Finally, two protons are removed from two more pyrrole nitrogens and an iron atom is inserted to make siroheme (Fig. 1a). In making heme via the canonical pathway, uro’gen III first undergoes decarboxylation of its acetyl groups before iron insertion^9^. Decarboxylation of the acetyl groups at C12 and C18 are the first steps to make heme or heme *d*_1_ from iron-containing siroheme^3^.

In *Salmonella enterica* serovar Typhimurium (*S. enterica*) and other proteobacteria-like *Escherichia coli*, a single, multifunctional enzyme, siroheme synthase (CysG), performs the final three steps from uro’gen III to siroheme^10,11^ (Fig. 1b). The N-terminal enzyme module, CysG^B^, is a three-domain bifunctional dehydrogenase/ATP-independent class III ferrochelatase^12^ that performs the final two steps of siroheme biosynthesis. The three domains include an N-terminal Rossmann fold that binds NAD^+^, an α/β dimerization domain, and a C-terminal α-helical domain—CysG assembles as a homodimer^13^. Together, the subunits form a domain-swapped dimer, where positioning of the Rossmann fold from one subunit over the helical domain from the opposing subunit creates a large cavity. At the back of the cavity, S128 can undergo phosphorylation and, when phosphorylated, dehydrogenation is slowed and chelation is inhibited^13^. The C-terminal enzyme module, CysG^A^, is homologous to a well-studied class of SAM-dependent uro’gen III methyltransferases (SUMTs) that catalyzes the methylations to generate precorrin-2^10,13^.

CysG^A^ and CysG^B^ are both homodimers but they do not share the same symmetry axis because of potential for asymmetry in the linker between the modules^13^. Although the large cavity between the Rossmann fold and the helical domain in CysG^B^ is suggestive of a porphyrin-binding site, we do not know how precorrin-2 is positioned for dehydrogenation at C15. In addition, despite extensive mutagenesis to identify the metal ligand^13,14^, it is unclear how iron is selected and inserted in the final step.

To answer these questions, we determined the X-ray crystal structures of *S. enterica* CysG bound to precorrin-2, sirohydrochlorin, and cobalt-sirohydrochlorin (co-sirohydrochlorin). A significant rotation that initiates at the dimer interface and is transmitted through an α-helix constrains the space between the Rossmann fold and the all-helical domain to specifically coordinate the tetrapyrrole for site-specific modification. Only one active site per homodimer binds substrate/co-substrate or product in any structure. Therefore, we used computational docking to simulate how NAD^+^/NADH would simultaneously bind with the co-substrate (precorrin-2) or product (sirohydrochlorin). Further, we rationally designed amino-acid variants to probe the specific function(s) of the numerous amino acids that bind the tetrapyrrole through its transition from sirohydrochlorin as product to substrate, and siroheme release.

## Results

### Tetrapyrroles bind between Rossmann fold and helical domain

*S. enterica* S128A-CysG was recombinantly expressed in *E. coli* and purified to homogeneity for crystallization and biochemical analysis. All experiments, including mutagenesis, were performed in the S128A background because wild-type CysG purifies with sub-stoichiometric phosphorylation at S128, and the S128A variant is more active for dehydrogenation and chelation than wild-type enzyme^13^. We refer to the sample as CysG throughout.

Anaerobic crystals were soaked with enzymatically prepared^15^ precorrin-2, sirohydrochlorin, or co-sirohydrochlorin. After 1–7 days, the crystals adopted the characteristic color of the substrate/product and this color persisted after backsoaking in cryogenic protective buffer. Crystals were frozen anaerobically and maintained at cryogenic temperatures for data collection. Phases were determined by molecular replacement with the CysG coordinates (PDB code 1PJQ^13^) for all tetrapyrrole-bound and amino-acid variant structures. A soak experiment with sirohydrochlorin and NADH resulted in bound sirohydrochlorin but no density for NADH. A soak experiment with precorrin-2 and NADH resulted in a structure with low-occupancy precorrin-2 bound to one active site but NADH, also at low occupancy, bound to the other.

**Fig. 2:**
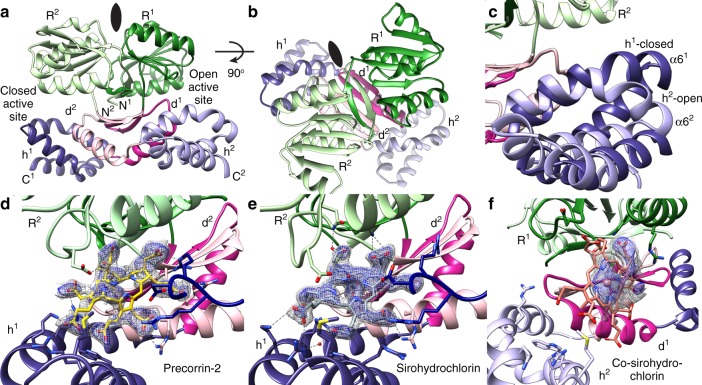
CysG^B^ architecture and tetrapyrrole binding. **a** CysG^B^ is a homodimer with asymmetric cavities between the Rossmann fold (R) of one subunit and the helical domain (h) of the other, joined by a dimerization domain (d). The N- and C-termini are labeled for each and the SUMT is not shown for simplicity. The domains and subunits are colored as in 1b. **b** The Rossmann fold and helical domains are orthogonal to one another. **c** The helical domain has a different conformation in the two subunits, resulting in one cavity that is closed and another that is open. **d** Precorrin-2 binds in the closed active site with strong density for the carboxylates but weaker density for the core ring. The tetrapyrrole binds with an average B-factor of 53.0 Å^2^ (compared with an overall B-factor for the structure of 50.9 Å^2^ and for local side chains of 51.9 Å^2^) and occupancy at 0.5. **e** Sirohydrochlorin binds to the closed active site with strong density. The tetrapyrrole binds with an average B-factor of 40.0 Å^2^ (compared with an overall B-factor for the structure of 51.3 Å^2^ and for local side chains of 48.6 Å^2^) and occupancy of 1.0. **f** Co-sirohydrochlorin binds to the open active site, nearly orthogonal to its conformation in the closed active site. The tetrapyrrole binds with an average B-factor of 77.3 Å^2^ (compared with an overall B-factor for the structure of 77.2 Å^2^ and for local side chains of 78.3 Å^2^) and occupancy at 0.5. All Polder omit *f*_o_–*f*_c_ maps are shown at 2σ (gray) and 3σ (blue).

The CysG^B^ homodimer is a domain-swapped dimer, with a large cavity between an NAD(H)-binding Rossmann fold from chain 1 and an all-helical domain from chain 2 (Fig. 2a). The domains cross in an α/β dimerization domain where two strands from each subunit make a four-stranded anti-parallel β-sheet that sits on top of the pair of helices. The Rossmann folds and all-helical domains are orthogonal to one another (Fig. 2b). Although the active sites are related by a twofold rotation, they are not identical. Specifically, in subunit 1, α-helix 6 from the all-helical domain is straight, making a closed active site. In subunit 2, α-helix 6 has a large bend at G159 that opens the active site by ~10 Å (Fig. 2c). Precorrin-2 and sirohydrochlorin were each bound to the closed site, whereas co-sirohydrochlorin was bound to the open site (Fig. 2d–f and Supplementary Movies 1–3).

Precorrin-2 is less oxidatively stable than more highly conjugated tetrapyrroles, and this was apparent in the weaker density of the central ring and partial occupancy of the molecule as a whole (Fig. 2d). On the other hand, density for the sirohydrochlorin was clearly defined (Fig. 2e). Co-sirohydrochlorin was not bound deeply in the open cavity so its density was more diffuse than that for the other tetrapyrroles, but sufficient to assign the central plane of the tetrapyrrole as nearly orthogonal to precorrin-2 and sirohydrochlorin (Fig. 2f). Fe^2+^ is insoluble in the crystallization and assay conditions described below, so Co^2+^ was used because CysG is also a sirohydrochlorin cobaltochelatase^16^, although selective for iron^11,17^.

### Numerous contacts between the tetrapyrrole and subunits

**Fig. 3:**
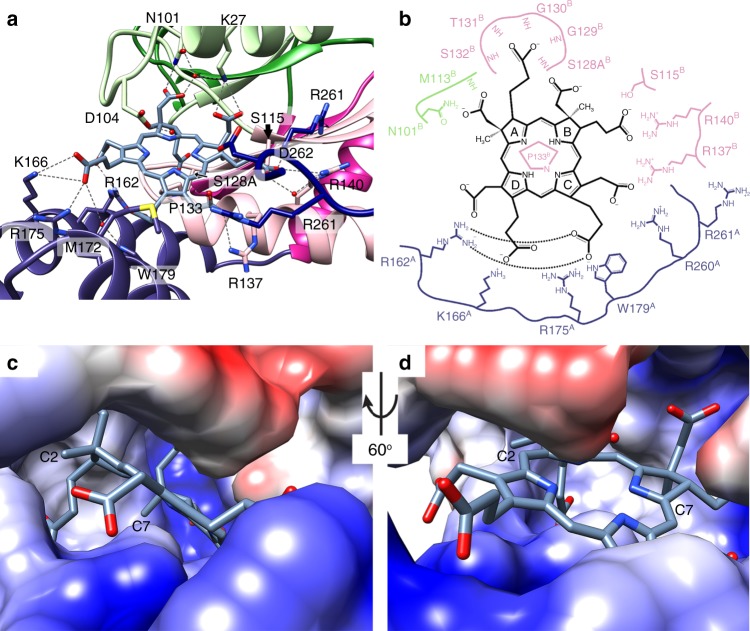
Charged pockets bind the tetrapyrrole. **a** Extensive contacts coordinate sirohydrochlorin. **b** Charge–charge side chain and hydrogen bond main chain interactions coordinate each corner of the tetrapyrrole. **c**, **d** The active site is predominantly positively charged (blue) to counter the highly negatively charged sirohydrochlorin, except for the negative charges from D104 and D262 (red) and hydrophobic pockets that position C2 and C7. The electrostatic surface was calculated with UCSF Chimera’s Electrostatic Surface Color^38^ tool.

The tetrapyrrole scaffold in siroheme precursors is highly negatively charged because of the eight acetyl and propionyl carboxylates, one of each at each corner. Each of these carboxylates interacts with arginine, lysine, asparagine, or serine side chains that make pockets for each of the pyrrole rings (Fig. 3a, b). Of the 12 amino acids that contribute polar or charged side chain hydrogen bonding or charge–charge interactions to bind sirohydrochlorin, four come from the subunit that contributes the Rossmann fold from subunit 1, and five from the all-helical domain from subunit 2. (To keep track of which amino acid derives from which subunit, we use a numerical superscript 1 or 2 after the amino-acid number.) Two arginines derive from CysG^A^, the SUMT (methyltransferase) module, one of which directly interacts with the siroheme (R260^1^) and one of which contributes to the electrostatics of the binding site but is too far away to directly interact (R261^1^, Fig. 3a).

Precorrin-2 and sirohydrochlorin make similar interactions between their carboxylates and positively charged and polar amino acids; described here are CysG’s interactions with sirohydrochlorin (Fig. 3a, b). The ring A C2 acetyl forms a hydrogen bond to N101^1^Nδ and a water-mediated hydrogen bond with N101^1^Oδ. The ring A C3 propionyl is 4.0 Å away from amino acid 128; modeling a serine shows that it would form a hydrogen bond with that propionyl if not modified (Supplementary Fig. 1a). When modified with a phosphate group, or with a tightly bound sulfate as in the CysG^B^ homolog from yeast, Met8p^14^, the negatively charged group would sterically inhibit the ring A C3 propionyl group from binding (Supplementary Fig. 1b). In the structure of phosphorylated S128-CysG^13^, an acetate molecule from the crystallization conditions binds near, but not exactly at, the ring B C7 acetyl position, which forms a bidentate interaction with K27^1^Nζ (Supplementary Fig. 1b). A water-mediated hydrogen bond links the ring B C8 propionyl to R140^1^Nη (Fig. 3a, b). The ring C C12 acetyl coordinates R137^1^Nη and R260^2^Nε and η. The ring C C13 propionyl coordinates both R162^2^Nη nitrogens in a bidentate manner as well as making a water-mediated hydrogen bond with W179^2^Nε, an interaction that is allowed because of the straightened helix 6. The ring D C17 propionyl coordinates K166^2^Zζ and R175^2^Nη. Finally, the ring D C18 acetyl coordinates both the R162^2^Nε and Nη. M172 flips over, serving as a hydrophobic block across the space between the Rossmann fold and all-helical domain at ring C. Overall, the binding pocket is highly charged with specific coordination at each corner to anchor the tetrapyrrole deep within the bifunctional active site (Fig. 3c, d).

In addition to the extensive network of side chain polar and charged interactions, several backbone interactions contribute to binding (Fig. 3b). For example, the ring A acetyl forms a hydrogen bond with the amide hydrogen from M113^1^. The ring A propionyl forms extensive hydrogen and water-mediated hydrogen bonds to the amide hydrogens on A128^1^, G130^1^, and S132^1^. The ring B propionyl participates in a water-mediated hydrogen bond with the amide hydrogen from I118^1^. The ring C propionyl forms a water-mediated hydrogen bond with V134^1^.

### Hydrophobic pockets orient the ring amid charge interactions

The methyl groups at positions C2 and C7 add additional features to the pattern of acetyl and propionyl substituents, selecting for the bismethylated intermediate over more highly methylated precursors to the related corrin ring in vitamin B12. G130^1^ sits on a loop 3.9 Å away from the C2 methyl group and A136^1^Cβ sits 3.9 Å away from the C7 methyl group, creating hydrophobic pockets for these smaller substituents (Fig. 3c, d).

### CysG^A^ contributes residues important to CysG^B^’s activities

**Fig. 4:**
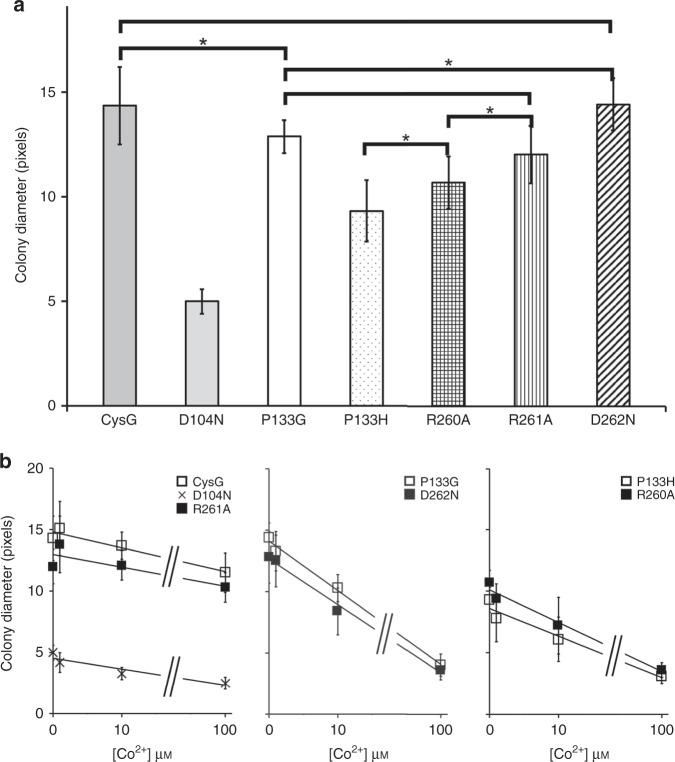
CysG variants respond differently to cobalt challenge. **a** ANOVA pairwise comparison in a two-tailed test of CysG-deficient *E. coli* expressing each variant (*n* = 18, CysG; = 37, D104N; = 21, P133G; = 25, P133H; = 18, R260A; = 18, R261A, = 15, D262N) was used to show which were statistically impacted by growth on M9 media. All were different than one another with a *p* value of < 0.001 (see Supplementary Table 1 for details) except those marked here. “*”≤ 0.5 and statistically indistinguishable. There was a significant effect of the CysG variant with an *F* value of 168.5 and 6 degrees of freedom. Error bars show standard deviations. **b** ANCOVA analysis of the impact of increasing cobalt concentration on colony size for CysG-deficient *E. coli* expressing each variant, interpreted as the slope of the line shown in each graph, followed by ANOVA analysis of the slopes to assess whether each variant was affected in a similar or different way. There was a significant interaction between each CysG variant and cobalt concentration with an *F* value of 43.1 and 6 degrees of freedom (number of colonies counted for each Co^2+^ concentration provided in Supplementary Table 2). Three groups arose: those that were affected like CysG (D104N and R261A), those that were dramatically affected (P133G and D262N), and those that were more modestly affected (P133H and R260A). Error bars show standard deviations. Source Data are provided as Source Data File SourceData_co-competition.txt.

R260, which binds ring C C12 acetyl, and R261, which does not directly interact with carboxylate but contributes to the positively charged pocket that captures ring B, are part of the CysG^A^ SUMT module. To test whether or not their contributions are essential for tetrapyrrole binding, we individually altered each to an alanine and tested the variants’ activities for dehydrogenase or chelatase activity. Both the R260A and R261A variants complement a cysG deficiency in *E. coli* grown on minimal media where SO_4_^2−^ is the sole sulfur source (Table 1). No Fe^2+^ is added to the media so *E. coli* scavenges Fe^2+^ to support growth in the S128A background. Interestingly, the culture expressing R260A, but not R261A, showed signs of precorrin-2 buildup in its pink fluorescence (Table 1). R261A responded similarly to CysG to Co^2+^-challenged complementation, whereas R260A colonies were smaller and more strongly affected by increasing Co^2+^ concentration (Fig. 4 and Supplementary Table 1).

**Table 1:** Complementation and activity assays.

CysG variant	*cysG*^*−*^		Dehydrogenase activity	Cobalt chelatase activity
	Fluorescence	Complementation	(nmol precorrin-2 min^−1^ (mg CysG)^−1^)	(nmol sirohydrochlorin min^−1^ (mg CysG)^−1^)
S128A		+ + +	47.4 ± 4.4	203 ± 3
D104A	+ + +	−	0	0
D104N	+ + +	+	3.3 ± 0.9	1.5 ± 0.5
P133G	+	+ +	2.7 ± 0.2	14.2 ± 1.3
P133H	+	+	0	19.2 ± 0.5
R260A	+ + +	+	0	3.1 ± 0.1
R261A	−	+ + +	1.4 ± 0.6	13.6 ± 0.6
D262A	+ +	+ +	20.3 ± 3.1	4.7 ± 0.5
D262N	+ +	+	2.5 ± 0.1	3.4 ± 0.4

+ + + , complements well; + + , complements; + , complements poorly.

Activity assays showed that the R260A variant is deficient for dehydrogenase activity and ~100-fold less active as a chelatase. The R261A variant has ~40-fold lower dehydrogenase activity and 20-fold lower chelatase activity. Non-enzymatic oxidation of precorrin-2 is sufficient to support complementation of a dehydrogenase-deficient but chelatase-active enzyme^17^, explaining why R260A complemented despite deficiency for dehydrogenase activity.

We next determined the X-ray crystal structures of the variants to test whether these alterations changed the relationship between the enzyme modules or their overall structure. Both R260A and R261A structures are similar to CysG and have a low root-mean-square deviation (RMSD) when aligned against Met8p^14^ (Supplementary Fig. 2a and Supplementary Table 3). R261 does not make a formal interaction with the tetrapyrrole, although it does contribute to the charged pocket that binds the carboxylates from ring B (Fig. 3a), so it is not surprising that removing the large, charged side chain does not substantially affect the structure. R260 directly interacts with the ring C carboxylates, but removing the large side chain does not substantially alter CysG’s structure despite its participation in forming the active site (Supplementary Fig. 2a).

### Precorrin-2 and sirohydrochlorin bind similarly

The interactions between the acetyl and propionyl groups from precorrin-2 and sirohydrochlorin are highly similar (Fig. 2d, e). The major difference lies in the degree to which the ring is rigidly held in the cavity. Precorrin-2 is a highly oxygen-sensitive dipyrrocorphin that lacks the ring conjugation of the later, more stable, isobacteriochlorins, sirohydrochlorin and siroheme^18^. Consequently, like uro’gen III^19^, precorrin-2 is flexible, so density for the central ring is not as strong as for sirohydrochlorin. Nevertheless, we see similar changes to the conformation of the helical domain and surrounding loops that are associated with tetrapyrrole binding, so we were able to assign a binding position for precorrin-2 that positions C15 towards the Rossmann fold.

Sirohydrochlorin and siroheme are isobacteriochlorins composed of two pyrrole and two pyrroline rings. Consequently, the whole tetrapyrrole is not conjugated: C2, C3, C7, and C8 from rings A and B are sp^3^ conjugated (Fig. 1), allowing for the bent conformation of siroheme in the S/NiR active site^20^. Nevertheless, sirohydrochlorin is more rigid than precorrin-2 and that definition is reflected in the improved electron density for sirohydrochlorin (Fig. 2e). In sirohydrochlorin, two of the four pyrrole nitrogens are protonated and two have a lone pair of electrons. Although sirohydrochlorin is more planar than precorrin-2, it is bowed in such a way as to position the ring nitrogens towards a water molecule that is 2.3 Å above the plane of the tetrapyrrole nitrogens that also interacts with D104 (Fig. 3a).

### NAD(H) binding clashes with the tetrapyrrole-bound scaffold

**Fig. 5:**
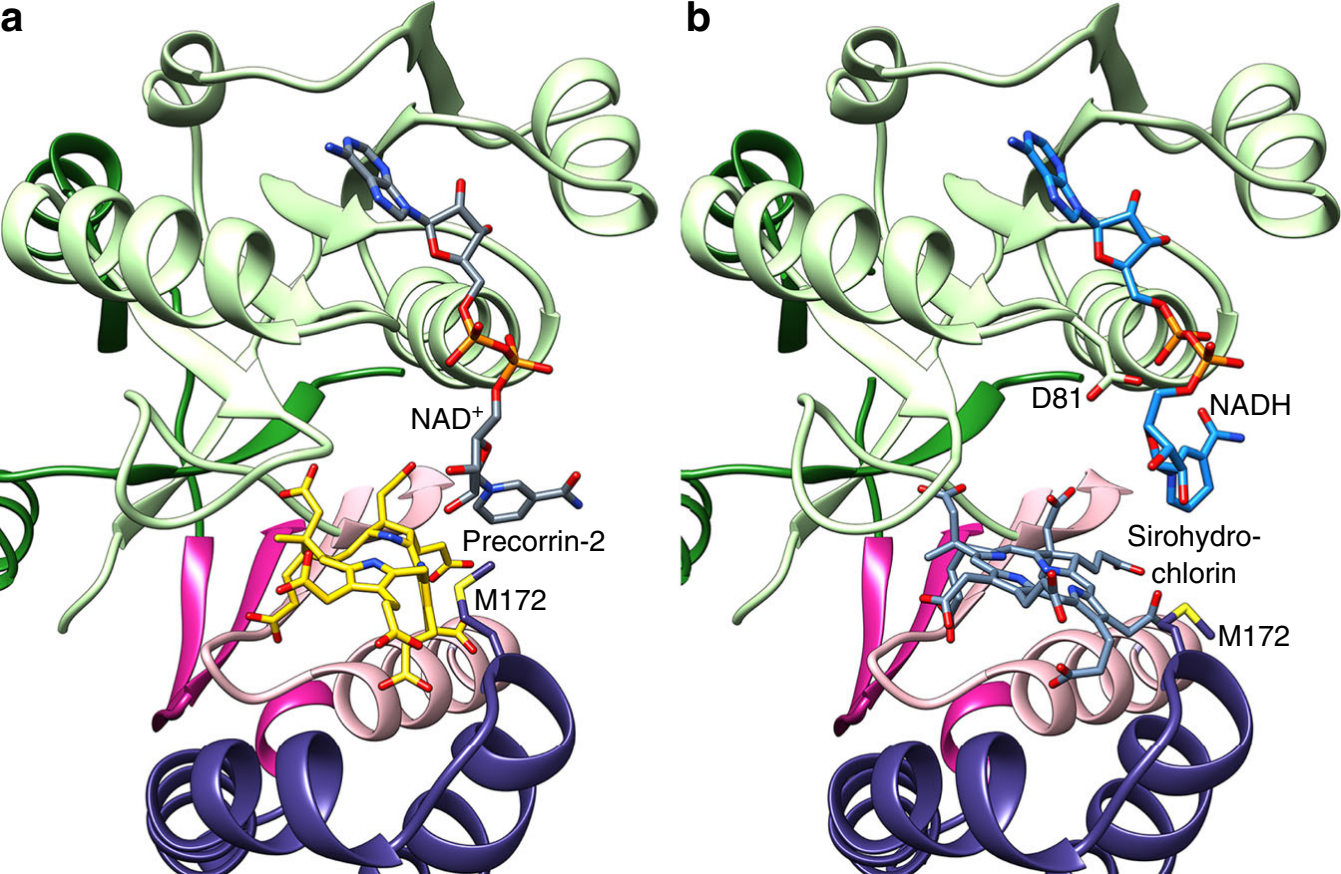
Docking of NAD(H). **a** Computational docking of the NAD^+^ (dark gray) to position the nicotinamide ring close to C15 in precorrin-2 (gold) required removing D81’s side chain and reorienting M172, which take on positions that are antithetical to NAD^+^ binding when the tetrapyrrole is present in the current crystal structure. The best binding pose for NAD^+^ is shown after those changes were made, demonstrating how the phosphates must make close approach for the nicotinamide ring to position itself and suggesting that some small conformational changes involving D81 and M172 may occur in solution. **b** Including D81 and M172 pushes the nicotinamide ring of the NADH (blue) away from the more conjugated sirohydrochlorin (gray) in the docked structure, perhaps explaining how the reaction proceeds after catalysis.

We used computational docking of NAD^+^ or NADH to the precorrin-2 or sirohydrochlorin-bound structures to clarify how the co-substrates might bind, because double-soak attempts did not result in binding to the same active site. The docking revealed details about interactions between the protein scaffold and NAD(H) phosphates/nicotinamide ring, explaining that experimental result. For example, in the apo structure, D81 points away from the active site cavity, toward the adenine-binding pocket of the Rossmann fold (Supplementary Fig. 3). In the precorrin-2 and sirohydrochlorin-bound structures, D81 is oriented toward the cavity (Supplementary Fig. 3), repelling the negatively charged NAD(H) phosphates and precluding close approach of the nicotinamide ring to the tetrapyrrole. In addition, the helical domain moves M172 to enclose precorrin-2 and sirohydrochlorin, but the large side chain then sterically blocks close approach of the nicotinamide ring (Supplementary Fig. 3). Removing the D81 side chain and repositioning M172 created a more favorable environment to computationally dock the NAD^+^ deeply in the pocket, pointing the nicotinamide ring toward C15, as it must for a productive interaction (Fig. 5a). In contrast, including the side chains in their sirohydrochlorin-bound positions revealed a separate binding pose for the NADH, where the phosphates flipped away from D81 and the nicotinamide ring pushed out from M172 (Fig. 5b). Computational docking of sirohydrochlorin as a control placed it at its experimentally determined position, supporting the accuracy of the docked poses (Supplementary Fig. 4).

### Co-sirohydrochlorin does not fit in the closed active site

We attempted to trap the co-sirohydrochlorin product by co-soaking sirohydrochlorin and cobalt. The resulting structure was lower resolution than the other structures and the closed active site that bound precorrin-2 and sirohydrochlorin showed no additional density. The open active site, however, contained a large, flat density that we interpreted as corresponding to a loosely bound product, sitting nearly perpendicularly to the position of the intermediates in the closed active site (Fig. 2f).

### P133 forms a platform for tetrapyrrole binding

**Fig. 6:**
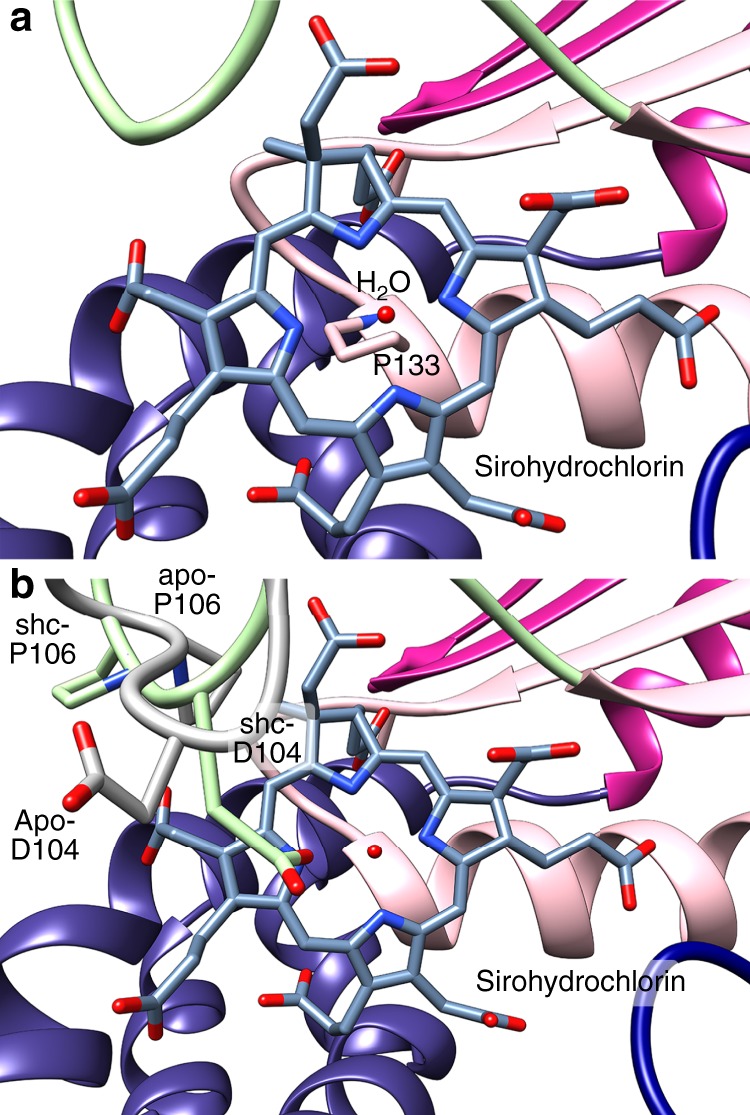
P133 and D104 are positioned below and above the sirohydrochlorin. **a** Sirohydrochlorin (dark gray) sits direction on top of P133 and below a single water (red sphere). **b** Upon sirohydrochlorin (dark gray) binding, the DAPK loop flips to position D104 directly over the pyrrole nitrogens, tightly coordinating the water molecule and P106, capping the tetrapyrrole. The apo position of the loop containing D104 (apo-D104) and P106 (apo-P106) is in light gray, whereas the sirohydrochlorin-bound loop containing D104 (shc-D104) and P106 (shc-P106) is in light green.

P133^1^Cβ sits 3.3 Å below the plane formed by the sirohydrochlorin isobacteriochlorin core, forming a hydrophobic platform for the macrocycle (Fig. 6a). Both P133G and P133H variants retained the ability to complement the *cysG*-deficient *E. coli* when challenged for growth on minimal media, albeit with accumulation of fluorescence indicating altered activity (Table 1). The P133G variant has 5-fold lower dehydrogenase activity and is about 10-fold lower in its chelatase activity, whereas the P133H variant is deficient for dehydrogenase activity and about 10-fold lower in its chelatase activity (Table 1). In the Co^2+^-challenge experiment, both variants show increased sensitivity to increasing Co^2+^ compared to CysG, with P133G more sensitive than P133H (Fig. 4 and Supplementary Table 1).

Structurally, the histidine side chain projects in the active site, where it would interfere with tetrapyrrole binding (Supplementary Fig. 2b), but no gross structural defects were present in the P133H variant as evidenced by a low RMSD to Met8p (Supplementary Table 3). In the P133G-closed active site that binds the tetrapyrrole, the first turn of the α helix from the dimerization domain is unwound, altering the hydrophobic platform on which the evolving substrate sits (Supplementary Figs. 2b and 5). The structure of the open active site, as well as the overall fold, is unchanged (Supplementary Figs. 2b, 5, and Supplementary Table 3).

### A loop flips to cap binding and put D104 above active site

A significant remodeling event occurs with tetrapyrrole binding when a loop including D104-A105-P106-K107 (DAPK loop) flips over to lock the tetrapyrrole in the active site (Fig. 6b). When P106 flips with the loop, it moves outward from its apo position, 5.5 Å away from C20 to make space for, and hold in place, the bulky sirohydrochlorin. Methylation at the C20 position creates precorrin-3, the next step in partitioning precorrin-2 toward vitamin B12^21^, so P106 may also serve as a steric block to prevent other similar tetrapyrroles from binding for iron insertion.

One longstanding question about CysG’s function is how it binds the metal ion for insertion. D104 has been implicated in its chelatase activity but its exact role is unknown^14^. Alteration to alanine results in an enzyme that cannot complement the *cysG* deficiency and is inactive for both dehydrogenase and chelatase activities (Table 1). Alteration to asparagine results in an enzyme with a severe growth defect, 10-fold lower dehydrogenase activity, and 200-fold lower chelatase activity (Table 1). The D104N variant is also sensitive to addition of Co^2+^, but with the same dose–response as CysG (Fig. 4 and Supplementary Table 1).

Interestingly, in the precorrin-2 bound structure, D104 is in two conformations whose occupancies correspond to the partial occupancy of the precorrin-2 molecule. In the sirohydrochlorin-bound structure, the DAPK loop is completely repositioned, placing D104 over the tetrapyrrole nitrogens (Fig. 6b). D104^1^Oδ makes a 2.8 Å-long hydrogen bond with the water molecule that is sitting directly above the tetrapyrrole nitrogens, joining D104^1^Oδ to the position where the iron will ultimately be coordinated by the tetrapyrrole nitrogens.

The D104A and D104N structures were determined to ensure that there were no gross structural defects that arose from the alterations that might impact their function (Supplementary Fig. 2c). Indeed, both altered enzymes were crystallized and the structures are largely similar to Met8p (Supplementary Table 3). The asparagine side chain is pointed away from the tetrapyrrole binding pocket, toward the Rossmann fold, as D104 does in the apo structure. Presumably, in the D104N altered CysG, the asparagine can reorient and partially act in the same capacity as the aspartic acid in catalyzing dehydrogenation and chelation. In the D104A structure, there is a slight movement of the DAPK loop in the closed, but not open, active site, highlighting the asymmetry of the active sites.

### D262 from CysG^A^ also influences activity

D262 comes from the CysG^A^ module, opposing D104 but adjacent to R260/R261 and sitting above the tetrapyrrole nitrogens. The position of the two aspartic acids was reminiscent of the histidines that are important in the class II cobaltochelatases CbiK/CbiX^22^ or the histidine-glutamate pair in human ferrochelatase, also a class II chelatase^23^. Therefore, we wanted to know if D262 from CysG^A^ was important for catalysis, so we altered it to an alanine or an asparagine (D262A or D262N). No gross structural defects were apparent (Supplementary Fig. 2d and Supplementary Table 3). Both complemented *cysG* deficiency, but the altered enzymes produced the fluorescent intermediate, with defects in the dehydrogenase and chelatase activities (Table 1). D262N was more affected by increasing Co^2+^ than CysG (Fig. 4).

## Discussion

Different organisms synthesize siroheme differently. Uro’gen III methylation is catalyzed by a dimeric SUMT homologous to SUMTs involved in vitamin B12 biosynthesis^10^. Dehydrogenation is catalyzed by a dimeric NAD^+^-dependent enzyme that is unique to siroheme synthesis^11^. In some organisms, this enzyme is a bifunctional class III ferrochelatase, which inserts iron to make siroheme^11^. Other organisms use a distinct class II ferrochelatase whose structure is similar to heme ferrochelatase and vitamin B12 cobaltochelatase^12,24^.

Proteobacteria-like *S. enterica* or *E. coli* use a single gene product to synthesize siroheme, CysG. CysG is encoded by a gene fusion between a C-terminal SUMT (CysG^A^) and an N-terminal bifunctional dehydrogenase/ferrochelatase (CysG^B^)^11,13^. Fungi like *Saccharomyces cerevisiae* use a separate SUMT (Met1p) and bifunctional dehydrogenase/chelatase (Met8p)^14,25^. Firmicutes like *Bacillus* use three enzymes: SirA, the SUMT; SirC, the monofunctional dehydrogenase that is homologous to CysG^B^ and Met8p; and SirB, the class II ferrochelatase^24,26^. CysG^B^ and SirB also function as sirohydrochlorin cobaltochelatases but are selective for iron^17,24^. Until now, no structures of CysG bound to any substrates, products, or metals have been determined to show the molecular mechanism behind that specificity. Here, we present structures of a bifunctional dehydrogenase/type III ferrochelatase bound to its substrates and a metallated product.

As predicted, CysG^B^-like dehydrogenase/ferrochelatases share an active site, despite the very different chemistries. Hydride extraction at C15 transforms the unconjugated, conformationally unrestricted precorrin-2 to a more highly conjugated, rigid, sirohydrochlorin. Consequently, although both tetrapyrroles coordinate positively charged amino-acid side chains with their eight carboxylates, the ring of the less rigid precorrin-2 is not as ordered as it is in the sirohydrochlorin.

The tetrapyrroles are bound so molecules are positioned for the chemistry to take place. Precorrin-2, oriented with the C2 and C7 methyl groups buried in hydrophobic pockets and coordinated by positively charged amino acids at each corner, presents C15 toward the Rossmann fold where a tightly docked NAD^+^ places its nicotinamide ring. The changes that occur to CysG upon tetrapyrrole binding are in opposition to tight NAD(H) binding, suggesting that NAD^+^ binding is transient to allow CysG^B^ to close around sirohydrochlorin before proceeding to metal insertion. Once flattened, residual distortions to the ring B and D pyrroles point to the D104-bound water, suggesting a water-mediated role for D104 as a general base to facilitate proton abstraction, allowing iron insertion.

D144, the analogous amino acid to D104, was previously identified as important for bifunctionality of Met8p^14^. In CysG, the D104A variant is catalytically inactive for both activities, supporting the idea that the side chain serves as a general base to facilitate proton abstraction in both reactions. The presence of a water molecule coordinated between the tetrapyrrole nitrogens and D104 at a 101^o^ angle suggests a second, non-exclusive possibility: D104 serves as the ligand to the metal after tetrapyrrole binding and re-orientation of the DAPK loop to position D104.

This possibility explains why there is no structure of a metal-bound CysG^B^-like active site, despite extensive efforts^13,14^, because chelatase activity requires that the tetrapyrrole bind first. Then, once metal is inserted, there would be steric overlap between the metal and P133, leading to product release, as captured in the weakly bound co-sirohydrochlorin. The plane of the nitrogens is 3.1 Å above the closest carbon of P133’s side chain. Fe^2+^ has an ionic radius of 1.92 Å; Co^2+^ has an ionic radius of 1.94 Å; and C’s ionic radius is 1.7 Å. There would be at least a 0.5 Å clash between the atoms, disrupting the position of the tetrapyrrole and breaking the extensive electrostatic interactions between the tetrapyrrole carboxylates and the positively charged amino acids.

Sequence conservation and mutagenesis supports this idea as it answers some conflicting data^13,14,26^. D104 is conserved in the bifunctional enzymes but not the monofunctional dehydrogenase SirC. In SirC, a serine in that position is not essential for dehydrogenase activity but alteration to an aspartic acid does not restore chelatase activity^26^. Looking adjacent to that position reveals a lack of conservation—the DAPK loop contains SSFS in SirC, but is conserved in Met8p. Perhaps, this sequence does not allow the loop to reorient, so without the steric block from the proline, the sirohydrochlorin product is freer to release from the active site after NAD^+^-driven hydride abstraction. Further, D144A Met8p co-purifies with a fluorescent molecule, likely precorrin-2^14^. Perhaps, in this case, the tetrapyrrole binds, but without loop closure/metal insertion, the reaction cannot proceed and so product is not released by steric clash of the metal and P133.

D104A CysG is deficient in both dehydrogenase and ferrochelatase activities, whereas D104N activity is only partially reduced. Both D104 variants show buildup of a fluorescent intermediate, owing to the active SUMT. Asparagine rarely serves as a nucleophile, but there are some examples of proteases that use an asparagine in this capacity^27^, so it may be sufficient to allow basal catalysis. It may also be sufficient to coordinate the water molecule that is poised over the tetrapyrrole nitrogens to prepare the tetrapyrrole for metal insertion, albeit less efficiently than the aspartate. Alternatively, SirC proceeds without a nearby base^26^, leaving the dehydrogenation reaction to rely solely on NAD^+^, so perhaps CysG^B^ can also perform basal dehydrogenase catalysis by a similar mechanism. As regards metal ion specificity, however, D104N is no more sensitive to increasing cobalt dose than CysG, suggesting that metal ion specificity does not depend entirely on the nature of the ligand that recruits the metal to the tetrapyrrole.

Both P133 variants show deficient or diminished dehydrogenase/ferrochelatase activity and increased sensitivity to cobalt. Removing the proline platform makes the active site less rigid by unfolding helix 6, presumably impacting its ability to bind and orient the unrestricted precorrin-2 molecule. The bulkier histidine projects into the active site, positioned in place of the pyrrole nitrogens when the tetrapyrrole binds, so would also disrupt tetrapyrrole binding and release.

Surprisingly, R260, R261, and D262, which all originate in the CysG^A^ module so are not present in Met8p, make up the back side of the active site pocket. The R260A variant was deficient for dehydrogenase activity and significantly reduced for metal insertion. R261A, D262A, and D262N retain some activity for both chemistries. It is possible that the contribution of these amino acids from the SUMT module provides evolutionary benefit from the complicated gene fusion, resulting in a homodimer with two separate enzyme modules.

D262 is positioned over the tetrapyrrole, opposing D104, similar to how two histidines are positioned over co-sirohydrochlorin in CbiK/CbiX^22^ or a histidine and a glutamate are positioned over lead-protoporphyrin in ferrochelatase^23^. Interestingly, D104 and D262 interact with the same face of the sirohydrochlorin as the histidines in CbiK/CbiX (opposite the methyl substituents), but the ring is rotated 180^o^ in the pocket. In CbiK/CbiX, the ring C/D edge of the sirohydrochlorin is buried, whereas the ring A/B edge points to solvent^22^. In CysG, the ring A/B edge is buried so the ring C/D edge points to solvent, positioning C15 under the Rossmann fold. Given that both D262 variants retain activity, however, and its absence from Met8p, we conclude that in CysG, D262 is not strictly required. It does, however, interact with an arginine that directly binds the sirohydrochlorin, so helps form the active site to bind the tetrapyrrole in the correct orientation.

P133G/H, R260A, and D262A are more sensitive to increasing cobalt concentration than D104N, R261A, or CysG. P133G behaves similarly to D262N: strongly affected by increasing cobalt concentrations. P133H behaves similarly to R260A, somewhat less sensitive. P133, R260, and D262 are distinct from D104 and R260 in that they impact tetrapyrrole binding, whereas D104 has a role in the chemistry and R261 has a general role in affecting the charge environment. This suggests that metal ion specificity is influenced by the precise binding position of the tetrapyrrole, rather than the nature of the metal ligand(s), even if catalysis still occurs in a sub-optimal active site.

This conclusion is supported by a recent study that showed for CbiK, the *S. enterica* class II cobaltochelatase involved in vitamin B12 synthesis, metal ion specificity is determined by the relative free energies for metalation of the cellular cobalt buffers versus the chelatase, rather than the affinity of the metal ion for the protein^28^. Metal ions are not found free in the cell^29^, so correct partitioning from their buffers to their targets depends on the free energy for metalation being more favorable for the metalloprotein than the buffer^28^. In the case of CysG, the binding position of sirohydrochlorin in the pocket is optimized for iron over cobalt, depending on a number of interactions including those with P133, R260, and D262.

**Fig. 7:**
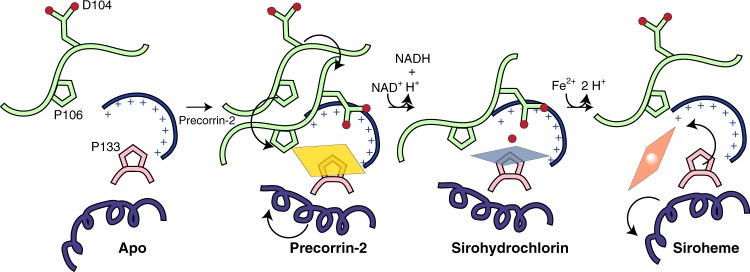
CysGʼs bifunctional dehydrogenase/chelatase active site. Model for the evolving sirohydrochlorin dehydrogenase product to chelatase substrate, on to the final siroheme product.

In summary, we determined structures of CysG^B^ bound to its substrate precorrin-2, the product and substrate sirohydrochlorin, and metallated co-sirohydrochlorin to explain how this unusual bifunctional enzyme catalyzes such different chemistries (Fig. 7). Both intermediates bind to the closed active site, coordinated by extensive charge–charge interactions and hydrogen bonds, positioned over P133 and under D104, both of which are catalytically important. Interestingly, amino acids from the fused CysG^A^ SUMT module contribute to the tetrapyrrole binding, suggesting an evolutionary advantage of the chimeric enzyme over the individual enzyme modules. Given the differences between the binding poses of the unmetallated precorrin-2/sirohydrochlorin and the metallated tetrapyrrole, we propose a mechanism for product release: steric clash between the metal and the P133 platform induce the tetrapyrrole to break its extensive interactions with the binding pocket, allowing movement of helix 6 so the product can diffuse out of the active site. Positioning of the ring in the active site, as opposed to the nature of the metal ligand that directs chelation, has a role in metal ion specificity of the dual cobalto-ferrochelatase.

## Methods

### Materials and chemicals

All chemicals were purchased through Sigma-Aldrich unless otherwise stated.

### Cloning of CysG mutants

Amino-acid variants of CysG were created using a Phusion Site-Directed Mutagenesis kit (ThermoFisher Scientific, Waltham, MA, USA; primers given in Supplementary Table 4). All variants were cloned into DH5α cells and sequence verified by Eurofins Scientific (Jacksonville, FL, USA).

### Protein expression and purification

S128A *S. enterica* CysG was recombinantly expressed as an untagged protein from the pBAD vector in LMG194 *E. coli* cells. Cells were grown at 37 °C to an OD_600_ of 0.6 and induced with 0.05% *L*-Arabinose. After 4 h, the cells were harvested by centrifugation and the pellets resuspended in 65 mm potassium phosphate buffer, pH 8, with 200 mm KCl (65K200 pH 8.0). Cells were lysed using a microfluidizer (Microfluidics, Westwood, MA, USA). Lysate was clarified by centrifugation at 13,000 × *g* for 30 m and all centrifugation steps were performed in a 5810 R centrifuge equipped with a F-34-6-38 fixed angle rotor (Eppendorf AG, Hamburg, Germany). The clarified lysate was passed over a 5 mL Ni-NTA column on an AKTA Pure FPLC (GE Healthcare, Chicago, IL, USA) and eluted with 65K200 pH 8.0 buffer + 50 mm imidazole. (Untagged CysG has a weak affinity for Ni-NTA so was used as an initial purification step.) After elution, fractions were pooled and diluted 1:1 in dH_2_O before proceeding with anion-exchange and size exclusion chromatography^13^. The expression/purification procedure was repeated for each CysG variant.

### Structure determination

Crystals of S128A-CysG and the other variants were grown by hanging drop vapor diffusion at 20 mg/mL in 100 mm MES pH 5.0, 500 mm NaCl, 1% β-mercaptoethanol and 10–20% PEG4000. Crystals were crushed and microseeded into 4–6% PEG4000-producing large, diffraction-quality crystals after 7–10 days.

### Substrate biosynthesis

Precorrin-2 and sirohydrochlorin were produced by enzyme-coupled biosynthesis^15^. The pETcoco-2-*cobA-hemB-hemC-hemD* (ABCD) plasmid, which contains the four six-histidine-tagged enzymes required for precorrin-2 biosynthesis from 5-aminoleuvenic acid, was used to express the recombinant enzymes, subsequently purified over a Ni-NTA column. To produce sirohydrochlorin, pET14b-*sirC* was used to express *Bacillus megaterium* SirC and lysate from SirC-overexpressing *E. coli* was mixed with the ABCD enzyme lysate prior to Ni-NTA purification. The enzyme cocktail was then transferred to a Schlenk tube and degassed, on ice, under a vacuum for 10 min to remove oxygen. The degassed enzymes were then introduced into an anaerobic chamber (mBraun, Stratham, NH, USA) and passed over a G-25 desalting column equilibrated with degassed 50 mm Tris-HCl pH 8.0 + 100 mm NaCl (50T100 pH 8.0). The enzymes were then added to an amber vial containing 2 mg 5-aminoleuvenic acid, 1 mg SAM, and 1 mm fresh dithionite in 50T100 pH 8.0 and incubated at room temperature overnight. To produce sirohydrochlorin, 1 mg NAD^+^ was additionally added to the amber vial before overnight incubation.

### Anaerobic crystal soaking and freezing

Aerobically grown crystals were transferred to sitting drop trays and moved into the anaerobic chamber (mBraun). Crystals were immediately transferred to fresh sitting drops containing strictly anaerobic mother liquor and equilibrated overnight. Next, crystals were added to fresh, anaerobic drops containing either precorrin-2 + NADH, sirohydrochlorin + NADH, or co-sirohydrochlorin in 4% PEG4000. After 2 h in the dark, CysG crystals were back-soaked into fresh anaerobic mother liquor and frozen in loops. Diffraction data of anaerobic precorrin-2/NADH bound, sirohydrohlorin-bound, and co-sirohydrochlorin-bound S128A-CysG were collected at Argonne National Laboratory (ANL, Lamont, IL, USA) at the SER-CAT 22-BM or 22-ID beamlines with a 1 Å wavelength beam and at 293 ^o^K.

### Data collection and refinement

Diffraction data were indexed, integrated, and scaled using HKL2000^30^ and phased by molecular replacement using wild-type CysG (PDB 1PJQ^13^) as a search model in PHASER, as implemented in PHENIX^31^. Phases were refined in iteratively with the phenix.refine^31^ command followed by manual amino-acid substitutions, in the case of the amino-acid variants, and manual fitting in Coot^32^ (Supplementary Table 5). Ligands were given restraints with phenix.eLBOW^31^ using simple optimization and provided the unique 3-letter ligand codes PQ2 for precorrin-2 and SHN for sirohydrochlorin. Coordinates for each structure were deposited in the PDB; PDB IDs are listed in Supplementary Table 5. Ramachandran statistics are provided in Table 2. Density for each amino-acid variant confirms the altered amino acid (Supplementary Fig. 6).

**Table 2:** Ramachandran statistics for tetrapyrrole-bound and amino-acid variant CysG structures.

CysG variant	Favored (%)	Allowed (%)	Outliers (%)	Rotamer outliers (%)
Sirohydrochlorin-bound	97.9	1.7	0.2	0.2
Precorrin-2-bound	95.3	3.7	0.8	0.6
Co-sirohydrochlorin-bound	96.4	3.2	0.5	0.1
D104A/S128A	94.8	5.0	0.2	0.8
D104N/S128A	96.3	3.5	0.2	0.7
P133G/S128A	96.4	3.6	0.0	0.0
P133H/S128A	97.7	2.1	0.1	0.1
R260A/S128A	97.8	2.0	0.2	0.4
R261A/S128A	97.5	2.5	0.0	0.3
D262A/S128A	98.1	1.8	0.1	0.1
D262N/S128A	97.3	*2.5*	0.2	0.0

### In vivo complementation assays and cobalt challenge

A *cysG*-knockout Keio strain (JW3331)^33^ was transformed with either empty pBAD vector, pBAD-S128A-CysG, or pBAD-variant CysG. Cells were grown overnight in LB containing 100 µg/mL ampicillin and 50 µg/mL of kanamycin. Cultures were centrifuged at 4000 × *g* for 10 min and resuspended in 1× M9 salts, repeating three times to remove excess nutrients from the media. The cultures were normalized at OD_600_ and serially diluted 1:10 to a final dilution of 10^−7^. Cells were then plated with 5 µL from 10^−2^ to 10^−7^ on LB or M9 media containing 100 µg/mL ampicillin and 50 µg/mL of kanamycin and incubated overnight for 24–48 h. Each CysG variant was repeated in triplicate with controls on each plate.

### Statistical analysis

For the cobalt challenge experiment, each control or CysG variant-expressing JW3331 strain was grown and washed as described above. Cells were grown/processed as described above, then plated on M9 plates with 0, 1, 10, 100 mm CoCl_2_•H_2_O. We assessed growth by measuring colony size in Photoshop (Adobe, San Jose, CA, USA) using a digitized image of the plates. We evaluated the differences in variant growth in the absence of cobalt using analysis of variance to model the size of the surviving colonies as a function of the categorical variable (variant). We followed this test with a post hoc Tukey analysis to evaluate the significance of pairwise differences in colony size across the variants using the “multcomp” package in R^34^ (Fig. 4a and Supplementary Table 1). To test for differences in the response of each variant to cobalt concentration, we used analysis of covariance, modeling the size of surviving colonies as a function of a categorical variable (variant) and a continuous variable (cobalt concentration). In this analysis, the slope of the relationship between colony size and cobalt concentration represents the impact of increasing cobalt concentration on colony size. We again followed this test with a post hoc Tukey analysis to evaluate the significance of pairwise differences in slopes across the variants using the “multcomp” package in R^34^, using this analysis to group the variants based on the impact of cobalt on their survival, whether similar to CysG, heavily impacted, or modestly impacted (Fig. 4b).

### In vitro specific activity enzyme assays

Specific activity measurements were measured spectrophotometrically using an 8453 UV-Vis spectrophotometer in kinetics mode (Agilent Technologies, Santa Clara, CA, USA). Precorrin-2 dehydrogenase activity and sirohydrochlorin cobalt chelation were monitored by an increase or decrease of sirohydrochlorin concentration at an absorbance at 376 nm, respectively. All reactions were completed in anaerobic 10 mm Quartz septa-sealed cuvettes. The precorrin-2 dehydrogenase reaction contained 2.5 µm precorrin-2, 1 mm NAD^+^, and 1–10 µg of CysG in 50T100 pH 8.0. The sirohydrochlorin cobalt chelatase reaction contained 2.5 µm sirohydrochlorin, 20 µm CoCl_2_, and 1–10 µg of CysG in 50T100 pH 8.0. Reactions were started by injection of CysG enzyme and recorded concurrently with a blank to correct for any oxidation. Assays were repeated in triplicate for each of the CysG variants. Owing to the extreme air sensitivity of the substrates and the complex mixture that results from the enzyme-coupled synthesis, complete kinetic analysis was not feasible^26^.

### Computational docking of NAD^+^/NADH

Molecular docking was performed to evaluate the potential binding poses of NAD^+^ and NADH in the precorrin-2 complex and the sirohydrochlorin complex, respectively, using Dock 3.6 with procedures previous described^35–37^. In brief, for placing the NAD^+^/NADH in the active sites containing precorrin-2/sirohydrochlorin, matching spheres were generated based on superimposed NADH observed in the adjacent monomer of the crystal structure. A water molecule mediating the interaction between F65 and the adenosine moiety was also superimposed and treated as part of the receptor, together with the protein and tetrapyrroles. To enhance functionally relevant ligand conformation sampling and favor interactions with the residues observed to hydrogen bond with NADH in the crystal structure, partial charges were increased for some residues such as T44, G21, D22, and V23, particularly involving the backbone amide groups. In addition, multiple protein side chain conformations were evaluated in various docking experiments owing to potential clashes, preventing the proper placement of the nicotinamide group. In particular, D81 appeared to restrict the conformation of the diphosphate linker, whereas M172 directly blocked the access of nicotinamide to the tetrapyrroles in the active site. For docking NAD^+^ to the substrate complex, a productive conformation of NAD^+^ was observed when the side chain of D81 was removed and M172 side chain was rotated away from the active site during docking. For docking NADH to the product complex, an alternative D81 side chain conformation was used to reduce the restraints on the flexibility of the diphosphate linker, whereas other residues remained unchanged compared with the crystal structure. The top docking results were manually examined to select the conformations most relevant to the proposed reaction.

### Reporting summary

Further information on research design is available in the Nature Research Reporting Summary linked to this article.

## Supplementary information

Supplementary Information

Peer Review

Reporting Summary

Description of Additional Supplementary Files

Supplementary Movie 1

Supplementary Movie 2

Supplementary Movie 3

## Data Availability

All raw data and plasmids are available from the corresponding author upon request. The source data for Fig. 4 are supplied as source data file. All structure factors and models were submitted to the RCSB with the following accession codes: sirohydrochlorin-bound: 6P5X [10.2210/pdb6P5X/pdb] precorrin-2-bound: 6VEB [10.2210/pdb6VEB/pdb] co-sirohydrochlorin: 6PEX [10.2210/pdb6PEX/pdb] D104A/S128A: 6P7C [10.2210/pdb6P7C/pdb] D104N/S128A: 6P7D [10.2210/pdb6P7D/pdb] P133G/S128A: 6PQZ [10.2210/pdb6PQZ/pdb] P133H/S128A: 6PR0 [10.2210/pdb6PR0/pdb] R260A/S128A: 6PR1 [10.2210/pdb6PR1/pdb] R261A/S128A: 6PR2 [10.2210/pdb6PR2/pdb] D262A/S128A: 6PR3 [10.2210/pdb6PR3/pdb] D262N/S128A: 6PR4 [10.2210/pdb6PR4/pdb]

## Source Data

Source Data
