# Supplementary material for: Siroheme synthase orients substrates for dehydrogenase and chelatase activities in a common active site
Source: Nat Commun. 2020 Feb 13;11:864. doi: 10.1038/s41467-020-14722-1 (PMC7018833; doi:10.1038/s41467-020-14722-1)
Supplement: Supplementary file 1 — Supplementary Information [file 41467_2020_14722_MOESM1_ESM.pdf]

Supplementary Information

Siroheme synthase orients substrates for dehydrogenase and chelatase activities in a common active site

Pennington, *et al*

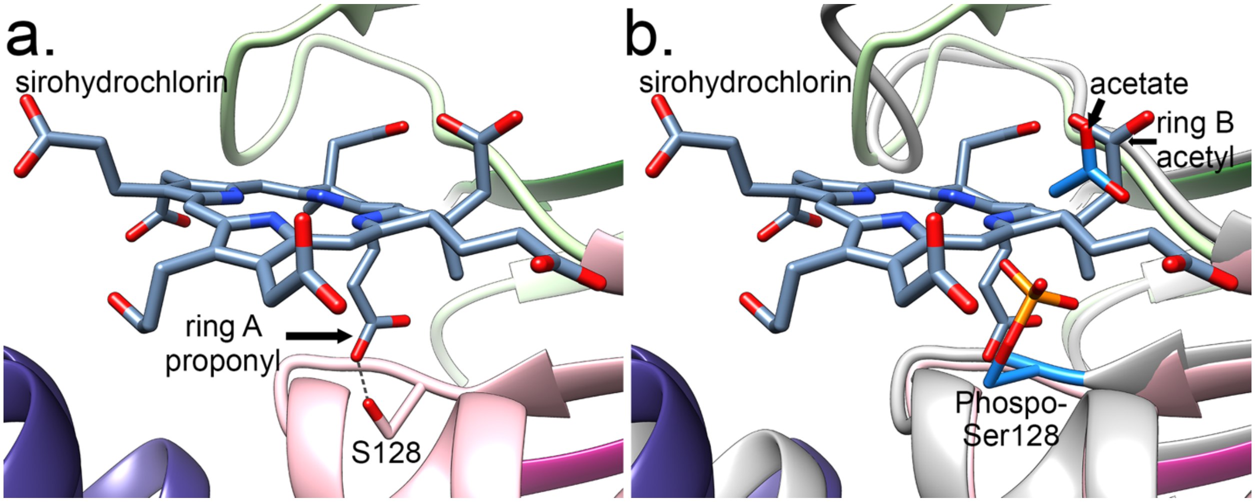

Supplementary Figure 1: *Sirohydrochlorin versus wild-type CysG.* **a.** Sirohydrochlorin would interact with S128 in the wild-type enzyme. **b.** The phosphoserine at position 128 would sterically clash with, and electrostatically repel, tetrapyrrole binding.

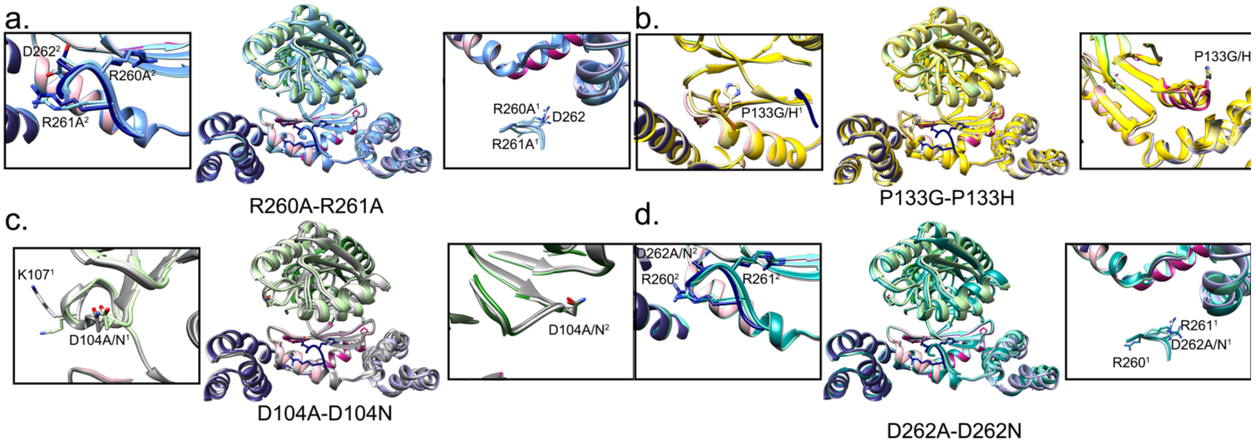

Supplementary Figure 2: *Amino acid variations do not dramatically alter CysG's structure.* The S128A single variant is colored as in all other figures in each panel. **a.** R260A (dark blue) and R261A (light blue). **b.** P133G (dark yellow) and P133H (light yellow). **c.** D104A (light gray) and S104N (dark gray). **d.** D262A (light cyan) and D262N (dark cyan).

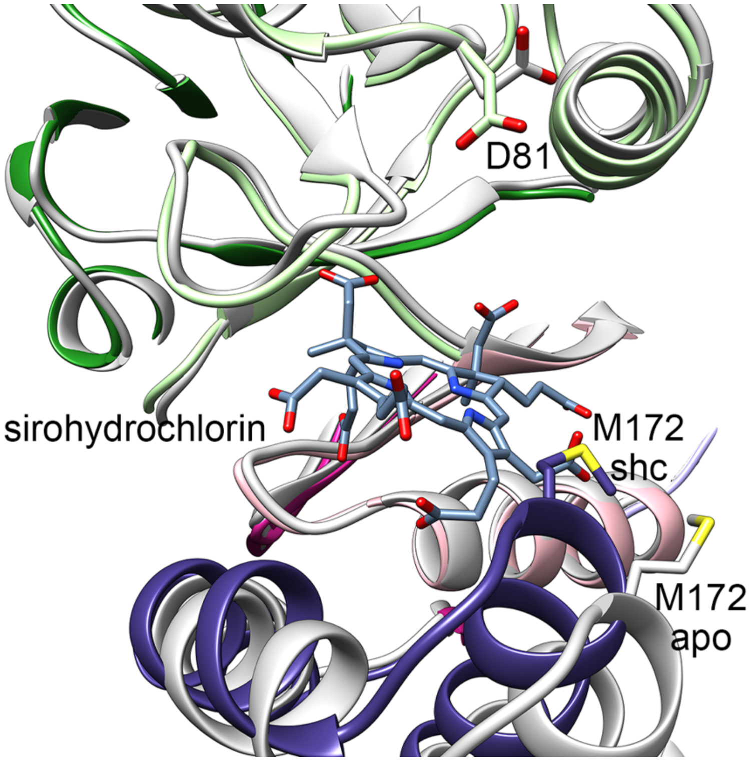

Supplementary Figure 3: *D81 and M172 undergo changes upon tetrapyrrole binding that are unfavorable for NAD(H) binding.* The apo structure (PDB ID 1PJS) is in white and the sirohydrochlorin-bound structure is colored as in all other figures.

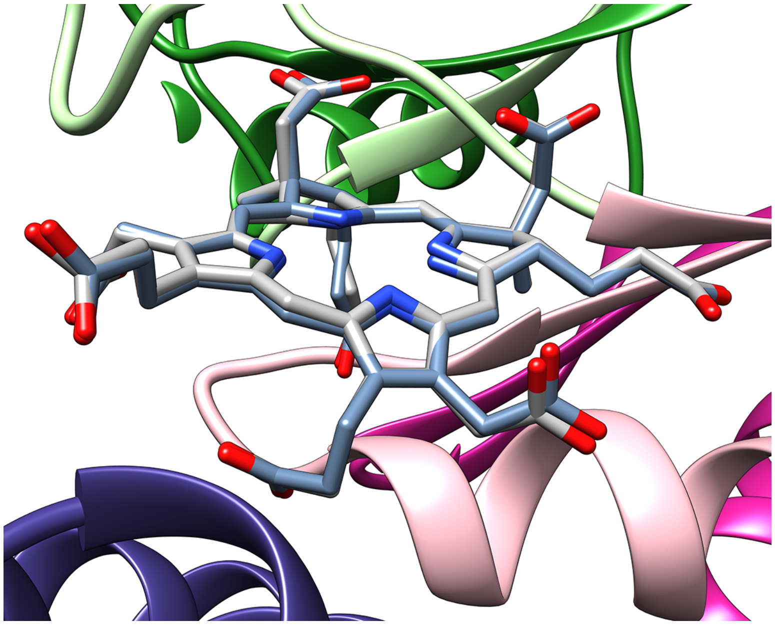

Supplementary Figure 4: *Computational docking of sirohydrochlorin (light gray) is very similar to the experimentally-determined position (dark gray).*

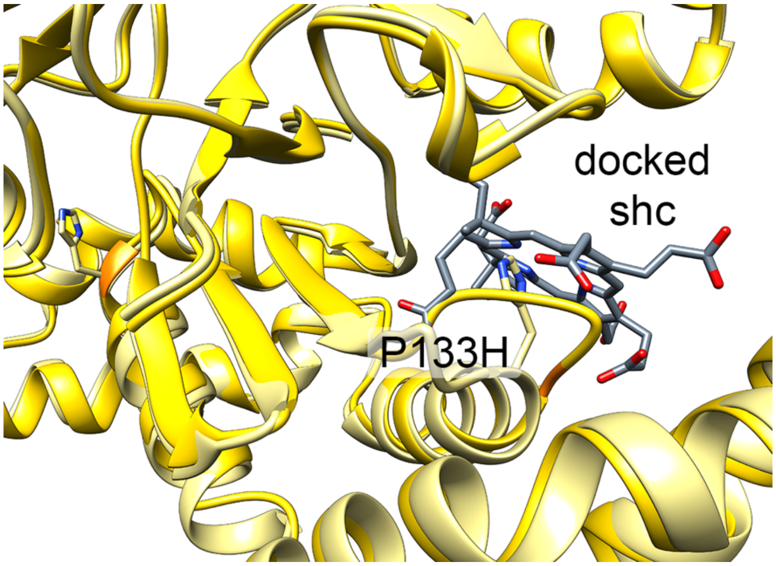

Supplementary Figure 5: *The P133G variant (darker yellow, orange residue is position 133), but not the P133H variant (lighter yellow, sidechain shown), unwinds the platform on which the sirohydrochlorin sits.* The histidine side-chain projects into the binding pocket. The sirohydrochlorin (shc) is docked to show how these changes might impact tetrapyrrole binding.

a. Sirohydrochlorin-bound CysG (Polder omit map at 2 (gray) and 3 (blue) σ)

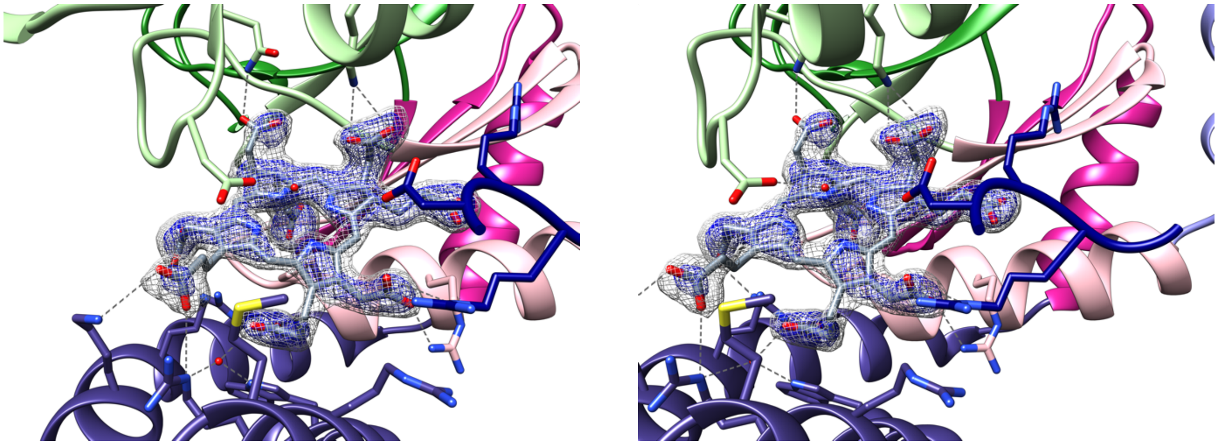

b. Precorrin-2-bound CysG (Polder omit map at 2 (gray) and 3 (blue) σ)

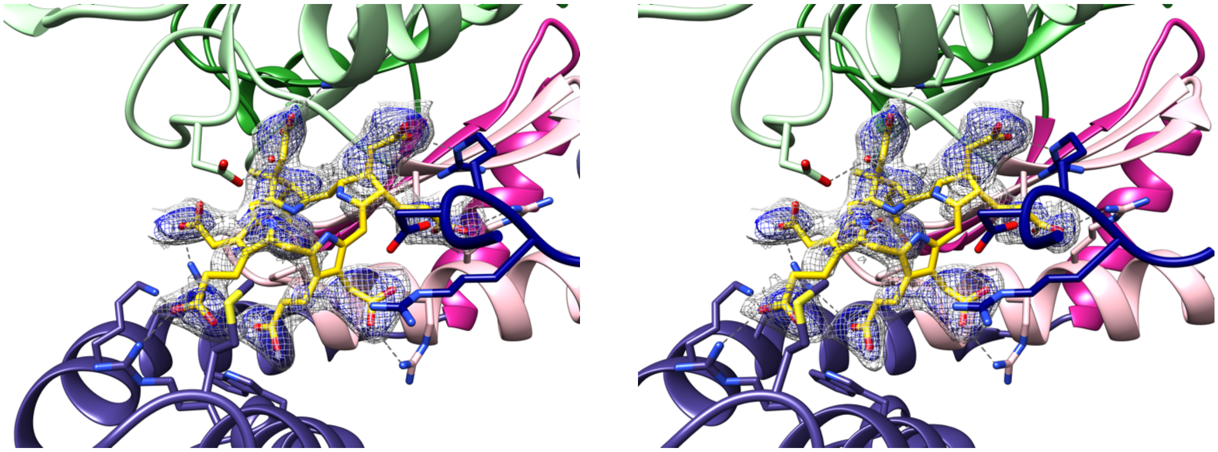

c. Co-sirohydrochlorin-bound CysG (Polder omit map at 2 (gray) and 3 (blue) σ)

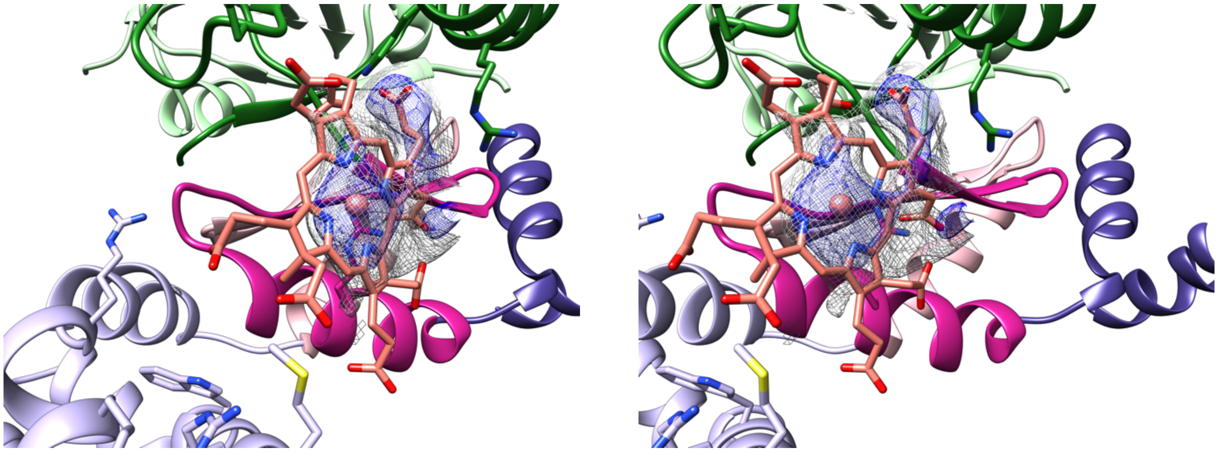

d.D104A (2mf_o-Df_c at 2 (gray) and 3 (blue) σ)

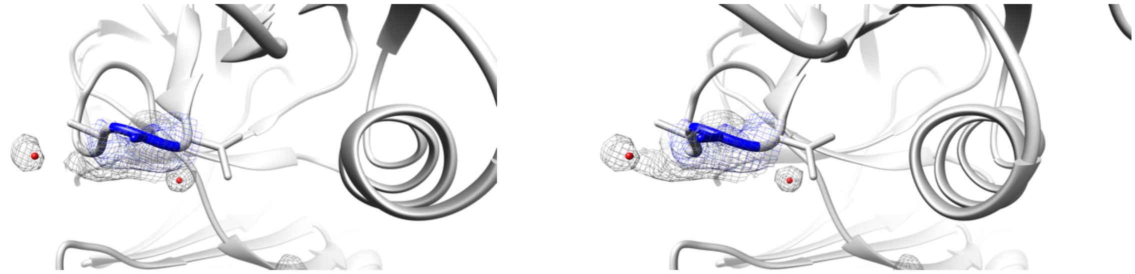

e. D104N (2mf_o-Df_c at 2 (gray) and 3 (blue) σ)

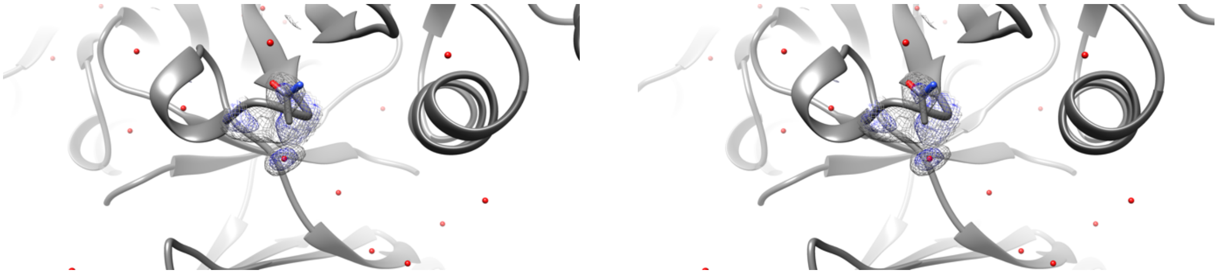

f. P133G (2mf_o-Df_c at 2 (gray) and 3 (blue) σ)

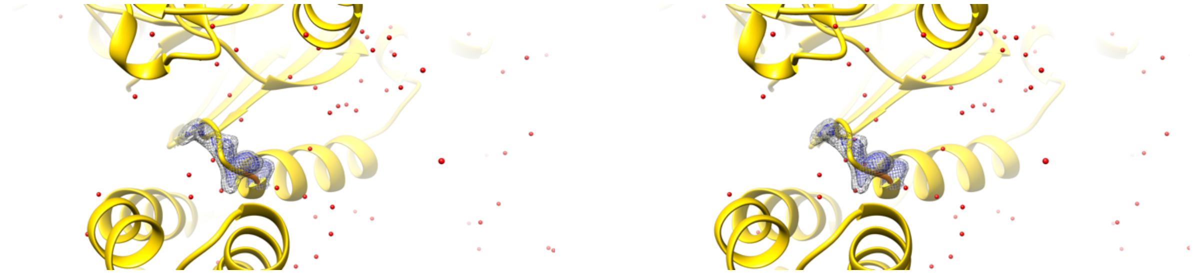

g. P133H (2mf_o-Df_c at 2 (gray) and 3 (blue) σ)

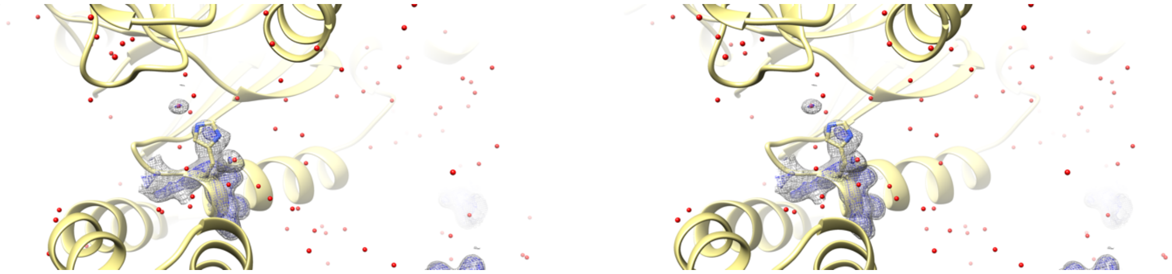

h. R260A (2mf_o-Df_c at 2 (gray) and 3 (blue) σ)

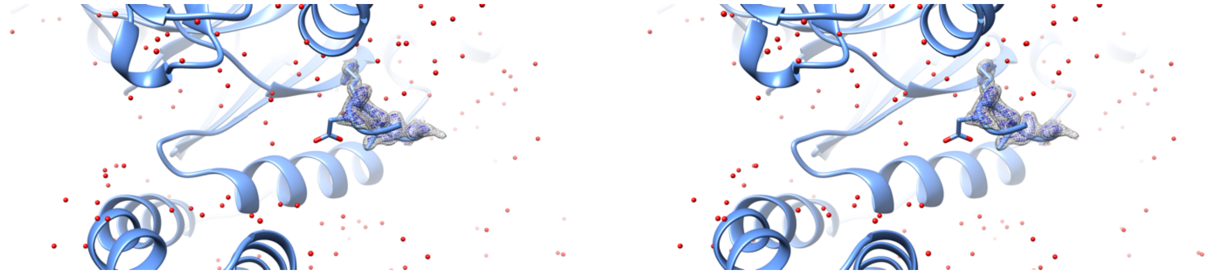

i. R261A (2mf_o-Df_c at 2 (gray) and 3 (blue) σ)

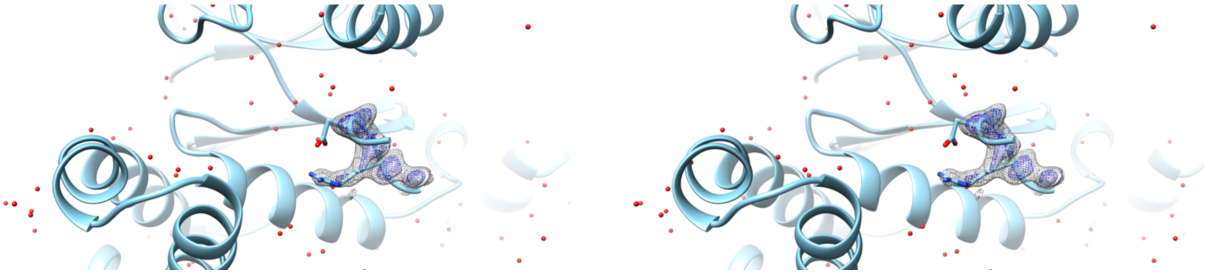

j. D262A (2mf_o-Df_c at 2 (gray) and 3 (blue) σ)

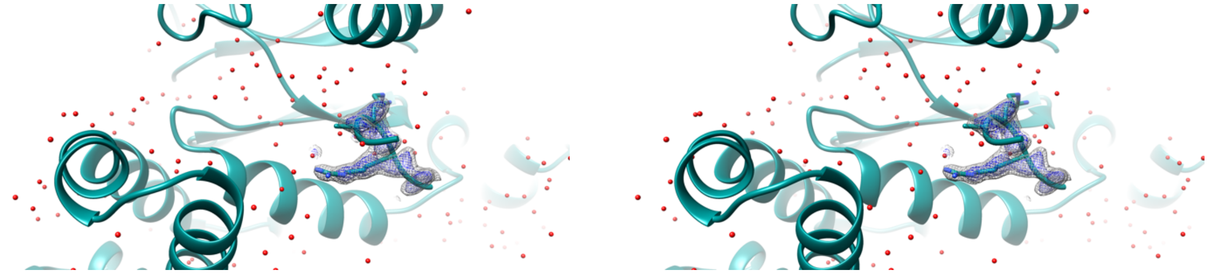

k. D262N (2mf_o-Df_c at 2 (gray) and 3 (blue) σ)

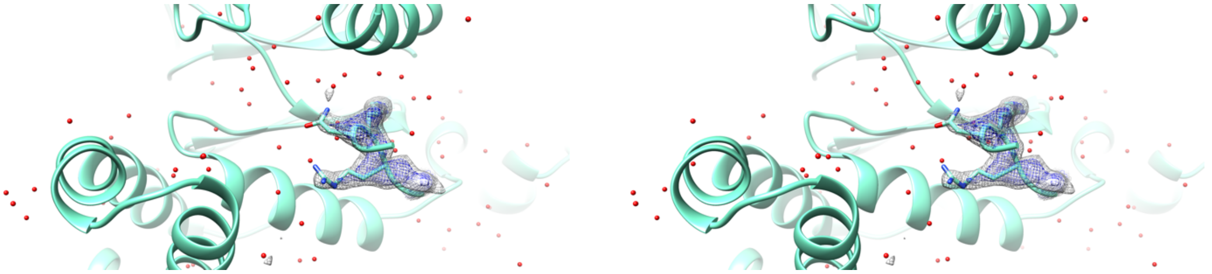

Supplementary Figure 6: *Stereoviews of each reported X-ray crystal structure.* a. sirohydrochlorin-bound CysG. b. precorrin-2-bound CysG. c. co-sirohydrochlorin-bound CysG. d. D104A CysG. e. D104N CysG. f. P133G CysG. g. P133H CysG. h. R260A CysG. i. R261A CysG. j. D262A CysG. k. D262N CysG.

Supplementary Table 1: p-values for pair-wise analysis of CysG amino acid variant growth on M9 minimal media

CysG variant pair	p-value for the null hypothesis
CysG-D104N	<0.001
CysG-P133G	0.01
CysG-P133H	<0.001
CysG-R260A	<0.001
CysG-R261A	<0.001
CysG-D262N	1
D104N-P133G	<0.001
D104N-P133H	<0.001
D104N-R260A	<0.001
D104N-R261A	<0.001
D104N-D262N	<0.001
P133G-P133H	<0.001
P133G-R260A	<0.001
P133G-R261A	0.4
P133G-D262N	0.01
P133H-R260A	0.02
P133H-R261A	<0.001
P133H-D262N	<0.001
R260A-R261A	0.05
R260A-D262N	<0.001
R261A-D262N	<0.001

Supplementary Table 2: Number of colonies for each specimen analyzed in Figure 4

	0 μM	1 μM	10 μM	100 μM
CysG	18	21	23	28
D104N	37	32	40	25
P133G	21	19	21	18
P133H	25	25	35	37
R260A	18	21	27	30
R261A	18	20	25	27
D262A	15	17	23	20

Supplementary Table 3: RMSD values for each chain of variant CysG^{B} against each chain of Met8p

CysG variant_chain ID	Met8p_chain ID	RMSD (Å)
CysG_a	Met8p_a	1.2
CysG_b	Met8p_a	1.2
CysG_a	Met8p_b	1.1
CysG_b	Met8p_b	1.1
D104A_a	Met8p_a	1.2
D104A_b	Met8p_a	1.1
D104A_a	Met8p_b	1.1
D104A_b	Met8p_b	1.1
D104N_a	Met8p_a	1.1
D104N_b	Met8p_a	1.1
D104A_a	Met8p_b	1.2
D104A_b	Met8p_b	1.1
P133G_a	Met8p_a	1.2
P133G_b	Met8p_a	1.2
P133G_a	Met8p_b	1.1
P133G_b	Met8p_b	1.1
P133H_a	Met8p_a	1.2
P133H_b	Met8p_a	1.1
P133H_a	Met8p_b	1.1
P133H_b	Met8p_b	1.1
R260A_a	Met8p_a	1.2
R260A_b	Met8p_a	1.2
R260A_a	Met8p_b	1.1
R260A_b	Met8p_b	1.1
R261A_a	Met8p_a	1.1
R261A_b	Met8p_a	1.1
R261A_a	Met8p_b	1.0
R261A_b	Met8p_b	1.1
D262A_a	Met8p_a	1.1
D262A_b	Met8p_a	1.2
D262A_a	Met8p_b	1.0
D262A_b	Met8p_b	1.2
D262N_a	Met8p_a	1.2
D262N_b	Met8p_a	1.2
D262N_a	Met8p_b	1.0
D262N_b	Met8p_b	1.1

Supplementary Table 4: Primers used for *cysG* mutagenesis

Primer Name	Primer Sequence (5' - 3')
D104A-StCysG-F	GCAACGTGGTGG**CT**GCGCCGAAAGC
D104A-StCysG-R	GCTTTCGGCGCA**G**CCACCACGTTGC
D104N-StCysG-F	CTTTTGCAACGTGGTG**A**ATGCGCCGAAAGCCG
D104N-StCysG-R	CGGCTTTCGGCGCAT**T**CACCACGTTGCAAAAG
P133G-StCysG-F	GGCGGCACCTCC**GGT**GTGCTGGCGCGTC
P133G-StCysG-R	GACGCGCCAGCAC**ACC**GGAGGTGCCGCC
P133H-StCysG-F	GCGGCACCTCCC**AC**GTGCTGGCGCG
P133H-StCysG-R	CGCGCCAGCAC**GT**GGGAGGTGCCGC
R260A-StCysG-F	CATTATGAACCTGGTA**GC**CCGCGATGCCGATCG
R260A-StCysG-R	CGATCGGCATCGCGG**GC**TACCAGGTTCATAATG
R261A-StCysG-F	GAACCTGGTACGC**GC**CGATGCCGATCGG
R261A-StCysG-R	CCGATCGGCATCG**GC**GCGTACCAGGTTC
D262A-StCysG-F	GGTACGCCGCG**CG**GCCGATCGGGTC
D262A-StCysG-R	GACCCGATCGGC**CG**CGCGGCGTACC
D262N-StCysG-F	CCTGGTACGCCGC**A**ATGCCGATCGGG
D262N-StCysG-R	CCCGATCGGCAT**T**GCGGCGTACCAGG

Supplementary Table 5a: Data collection and refinement statistics*

	sirohydrochlorin-bound**	precorrin-2-bound**	Co-sirohydrochlorin-bound**
**PDB ID**	6P5X	6VEB	6P5Z
**Data collection**			
Space Group	P 2_1 2_1 2_1	P 2_1 2_1 2_1	P 2_1 2_1 2_1
Cell dimensions			
a, *b*, *c* (Å)	60.1, 99.8, 145.5	59.7, 99.5, 146.0	59.6, 100.5, 146.4
α, β, γ (°)	90, 90, 90	90, 90, 90	90, 90, 90
Resolution (Å)	38.4 - 2.0 (2.04 - 1.97)	46.2 - 2.6 (2.64 - 2.55)	35.0 - 2.3 (2.40 - 2.30)
R_{merge}	0.103 (0.582)	0.268 (0.802)	0.0736 (0.636)
$I/\sigma I$	31.9 (5.2)	11.8 (1.6)	21.3 (2.2)
Completeness (%)	99.8 (99.5)	99.9 (99.7)	99.7 (97.9)
Redundancy	14.5 (12.0)	12.9 (6.2)	7.2 (5.9)
**Refinement**			
Resolution (Å)	38.4 - 2.0 (2.04 - 1.97)	46.2 - 2.6 (2.64 - 2.55)	35.0 - 2.3 (2.40 - 2.30)
No. of Reflections	62506 (6142)	29095 (2850)	39678 (3833)
R_{work}/R_{free}	0.174 (0.223)/0.221 (0.280)	0.173 (0.227)/0.246 (0.318)	0.17.8 (0.247)/0.242 (0.318)
No. atoms	7599	7493	7179
Protein	7283	7121	6929
Ligands	114	158	178
Water	314	207	68
R. m. s. deviations			
bonds lengths (Å)	0.007	0.009	0.008
bonds angles (°)	1.2	1.1	1.0
Average B-factor ($Å^2$)	50.9	50.9	77.7
macromolecules	51.3	50.9	77.6
ligands	38.7	53.0	87.9
SAH	37.3	33.1	56.6
tetrapyrrole	40.0	53.0	77.3
NADH	N/A	54.0	N/A
solvent	45.8	45.2	59.4

*Statistics for the highest-resolution shell are shown in parentheses.

**Single crystal used for data acquisition

Supplementary Table 5b: Data collection and refinement statistics*

	D104A/S128A**	D104N/S128A**	P133G/S128A**
**PDB ID**	6P7C	6P7D	6PQZ
**Data collection**			
Space Group	P 2_1 2_1 2_1	P 2_1 2_1 2_1	P 2_1 2_1 2_1
Cell dimensions			
a, *b*, *c* (Å)	59.9, 99.9, 147.5	60.1, 99.3, 147.4	59.3, 98.3, 143.2
α, β, γ (°)	90, 90, 90	90, 90, 90	90, 90, 90
Resolution (Å)	38.4 - 2.8 (2.85 - 2.76)	38.0 - 2.4 (2.48 - 2.40)	34.8 - 2.2 (2.31 - 2.23)
R_{merge}	0.491 (1.411)	0.230 (1.6)	0.188 (1.0)
$I/\sigma I$	17.6 (4.8)	18.6 (1.1)	19.1 (3.1)
Completeness (%)	99.5 (96.5)	99.9 (99.9)	98.9 (92.5)
Redundancy	12.5 (8.5)	12.4 (6.7)	6.9 (5.9)
**Refinement**			
Resolution (Å)	38.4 - 2.8 (2.85 - 2.76)	38.0 - 2.4 (2.48 - 2.40)	34.8 - 2.2 (2.31 - 2.23)
No. of Reflections	23509 (2263)	35283 (3460)	40938 (3752)
R_{work}/R_{free}	0.196 (0.271)/0.290 (0.385)	0.185 (0.273)/0.248 (0.366)	0.181 (0.249)/0.245 (0.320)
No. atoms	7077	7166	7189
Protein	7028	7053	7002
Ligands	52	52	52
Water	48	112	187
R. m. s. deviations			
bonds lengths (Å)	0.008	0.009	0.008
bonds angles (°)	1.3	1.3	1.3
Average B-factor (Å^2)	59.1	70.3	59.1
macromolecules	59.3	70.6	59.5
ligands - SAH	50.4	58.6	41.5
solvent	48.0	57.5	50.9

*Statistics for the highest-resolution shell are shown in parentheses.

**Single crystal used for data acquisition

Supplementary Table 5c: Data collection and refinement statistics*

	P133H/S128A**	R260A/S128A**	R261A/S128A**
**PDB ID**	6PR0	6PR1	6PR2
**Data collection**			
Space Group	P 2_1 2_1 2_1	P 2_1 2_1 2_1	P 2_1 2_1 2_1
Cell dimensions			
a, *b*, *c* (Å)	59.8, 99.9, 146.5	59.6, 99.6, 147.5	59.4, 99.8, 146.6
α, β, γ (°)	90, 90, 90	90, 90, 90	90, 90, 90
Resolution (Å)	38.3 - 1.9 (1.97 - 1.90)	47.2 - 1.8 (1.89 - 1.82)	41.2 - 2.2 (2.24 - 2.16)
R_{merge}	0.188 (1.2)	0.132 (1.29)	0.284 (2.06)
$I/\sigma I$	19.3 (1.8)	21.4 (1.5)	25.8 (4.4)
Completeness (%)	99.1 (98.5)	99.7 (97.3)	99.8 (99.7)
Redundancy	12.8 (8.9)	13.8 (8.2)	11.6 (9.2)
**Refinement**			
Resolution (Å)	38.3 - 1.9 (1.97 - 1.90)	47.2 - 1.8 (1.89 - 1.82)	41.2 - 2.2 (2.24 - 2.16)
No. of Reflections	69367 (6788)	79193 (7606)	47501 (4664)
R_{work}/R_{free}	0.180 (0.310)/0.230 (0.342)	0.175 (0.254)/0.208 (0.297)	0.166 (0.224)/0.216 (0.262)
No. atoms	7437	7603	7308
Protein	7044	7116	6982
Ligands	52	52	52
Water	393	485	311
R. m. s. deviations			
bonds lengths (Å)	0.008	0.007	0.008
bonds angles (°)	1.2	1.2	1.3
Average B-factor ($Å^2$)	48.7	43.8	56.0
macromolecules	48.7	43.7	56.1
ligands - SAH	32.1	27,9	40.9
solvent	49.3	46.1	54.8

*Statistics for the highest-resolution shell are shown in parentheses.

**Single crystal used for data acquisition

Supplementary Table 5d: Data collection and refinement statistics*

	D262A/S128A**	D262N/S128A**
**PDB ID**	6PR3	6PR4
**X-ray diffraction data**		
Space Group	$P\ 2_1\ 2_1\ 2_1$	$P\ 2_1\ 2_1\ 2_1$
Cell dimensions		
a, *b*, *c* (Å)	59.3, 98.9, 146.3	59.6, 98.8, 146.5
α, β, γ (°)	90, 90, 90	90, 90, 90
Resolution (Å)	35.2 - 2.0 (2.03 - 1.96)	38.0 - 2.2 (2.32 - 2.24)
R_{merge}	0.146 (0.926)	0.216 (1.09)
$I/\sigma I$	20.7 (3.3)	19.3 (2.5)
Completeness (%)	98.2 (89.1)	99.6 (96.6)
Redundancy	7.0 (4.9)	12.8 (6.2)
**Refinement**		
Resolution (Å)	35.2 - 2.0 (2.03 - 1.96)	38.0 - 2.2 (2.32 - 2.24)
No. of Reflections	61183 (5482)	42014 (3995)
R_{work}/R_{free}	0.168 (0.235)/0.214 (0.289)	0.168 (0.225)/0.226 (0.305)
No. atoms	7516	7324
Protein	7052	7063
Ligands	52	52
Water	462	261
R. m. s. deviations		
bonds lengths (Å)	0.008	0.008
bonds angles (°)	1.2	1.3
Average B-factor (Å^2)	47.8	55.7
macromolecules	47.6	56.0
ligands - SAH	33.2	40.8
solvent	52.2	52.3

*Statistics for the highest-resolution shell are shown in parentheses.

**Single crystal used for data acquisition
