# Supplementary material for: Siroheme synthase orients substrates for dehydrogenase and chelatase activities in a common active site
Source: Nat Commun. 2020 Feb 13;11:864. doi: 10.1038/s41467-020-14722-1 (PMC7018833; doi:10.1038/s41467-020-14722-1)
Supplement: Supplementary file 4 — Description of Additional Supplementary Files [file 41467_2020_14722_MOESM4_ESM.docx]

**Description of Additional Supplementary Files**

File name: Supplementary Movie 1
Description: Rotating view of the Polder omit map of the precorrin-2 density, corresponding to the view in Figure 2d.

File name: Supplementary Movie 2
Description: Rotating view of the Polder omit map of the sirohydrochlorin density, corresponding to the view in Figure 2e.

File name: Supplementary Movie 3
Description: Rotating view of the Polder omit map of the co-sirohydrochlorin density, corresponding to the view in Figure 2f.
