# Supplementary material for: Siroheme synthase orients substrates for dehydrogenase and chelatase activities in a common active site
Source: Nat Commun. 2020 Feb 13;11:864. doi: 10.1038/s41467-020-14722-1 (PMC7018833; doi:10.1038/s41467-020-14722-1)
Supplement: Supplementary file 2 — Peer Review [file 41467_2020_14722_MOESM2_ESM.pdf]

Reviewers' comments:

Reviewer #1 (Remarks to the Author):

The manuscript concerns investigations into the mechanism of CysG, the siroheme synthase multifunctional enzyme that transforms uroporphyrinogen III into siroheme. The enzyme performs methylations at C2 and C7 of the macrocycle before catalysing dehydrogenation and chelation reactions. The latter two reactions are performed in the N-terminal region of the protein within a single site. The authors have undertaken a significant amount of work including crystallography, mutagenesis and enzymology in order to try and compose a molecular overture of events to explain how this enzyme is able to perform the dehydrogenation and chelation reactions. Excitingly, the authors have managed to generate substrate and product complexes of the enzyme and from this are able to provide new insights into the action of the enzyme. This works makes a significant advance to the area and represents a fairly complete piece of work. The work complements on-going studies elsewhere that aim to understand how cellular metal availability is related to enzyme activity so this paper is rather timely. There are some comments the authors may wish to address below:

1. Throughout text, replace typhimurium with enterica. Alternatively, for this serotype, Salmonella enterica subsp. enterica serotype Typhimurium, it can be abbreviated to Salmonella Typhimurium. Personally, I'd stick with S. enterica.

2. In the abstract – it is stated "CysG's single active site … " but of course this is confusing. CysG has its methyltransferase active site, and is a dimer, so therefore technically there are 4 active sites??

3. I am not familiar with proponyl as a term – propionyl is the more traditional spelling. Also, vitamin does not require a capital V. On trivial points, the term porphine (P11) refers to a metal-free porphyrin, rather than an isobacteriochlorin.

4. It is stated on Page 3 – that siroheme is more like vitamin B12 in terms of having propionyl and acetyl side chains. Given that B12 is ring contracted – a better comparison would be with F430.

5. The results are well aid out and nicely explained. Given that the precorrin-2 density is so weak, I'm not sure that the authors can raw too much concerning its actual position and distortion – most of this is inferred. Similarly, I'm not sure they can, with real accuracy, say that the C15 position is located more closely located to the NAD than sirohydrochlorin, especially given that the NAD is modelled.

6. In the discussion, I'd point out that the CysG/Met8p chelatases represent a unique class III of chelatase, structurally distinct from the class I and II chelatases (see Brindley et al, 2003, J Biol Chem. 278, 22388-95).

7. I would try and compare the two aspartates (D104/262) found above the tetrapyrrole substrate in CysG to the residues found in CbiK/CbiX (two His; see Romão et al (2011), PNAS 108(1):97-102) and the ferrochelatases (His and Glu; Medlock et al 2007, PNAS 104(6):1789-93)

8. Finally, to put this back into a cellular context – I'd try and relate what you have found with the need to be able to bind Fe within the cell and relate this to the available intracellular metal concentration – see Osman et al (2019), Nat Chem Biol 15: 241-249.

Martin Warren

Reviewer #2 (Remarks to the Author):

The manuscript by Pennington et al. describes a very thorough study of the enzyme SiroHeme and its interactions with the changing prosthetic group. There are at least eleven diffraction and refinement data tables (Table 1 type reporting) with resolutions better that 2 Å and good refinement statistics.

The amount and the quality of information is outstanding. Nevertheless, it is difficult to present this amount of information in a way that makes it easy for the reader. This reviewer thinks that reading pages of results on eleven structures (or more) is difficult. The manuscript would be highly improved if the format is changed to make the section after the Introduction a Results and Discussion. Combining these two sections early in the manuscript will allow the reader to understand the changes described in the present Results section as they are presented as opposed to learn about them and their implications only after all observed changes are described..

Reviewer #3 (Remarks to the Author):

The manuscript presents X-ray crystal structures of Salmonella typhimurium siroheme synthase (CysG) liganded to substrates product and metals. CysG has a bifunctional active site that catalyzes the final two steps of siroheme production, NAD+-dependent dehydrogenation and iron chelation. Currently, it is not known how a single active site performs these different chemistries. Semi-quantitative assay of complementation and of the two activities are presented to test the importance of active site residues using site-directed mutagenesis. The topic of the manuscript should be of interest to a broad readership on enzyme mechanisms, iron utilization, and metabolic pathways. Generally, the manuscript is well written and the assay work informative but a lack of information for the X-ray crystallographic methodology makes it impossible to properly assess the technical merit. As detailed below, the authors should present debiased omit maps and information about the B-factors of the ligands in order to confirm the presence of the ligands, which are the basis for the findings. Indeed, the electron density that is presented for the structure of the complex with precorrin-2 appears very poor and raises serious concerns about this particular liganded structure. The authors might consider the following points (page numbers from PDF for review):

Major concerns:
1) The electron density shown in figure 2d,e,f is from 2Fo-Fc maps To adequately assess the ligand density a debiased polder or simulated annealing omit maps or composite omit maps should be shown here or in supplementary materials (and it would be best if they were in stereo view). Indeed, the density for the structure with precorrin-2 is extremely disconnected and although the authors discuss this point, without additional maps and information on temperature factors there cannot be confidence in the interpretation. The additional maps are of great importance - on the precorrin-2 global structure validation metrics from the PDB validation report are below average for the resolution in all but one category. Omit maps and the average B-factor of the precorrin-2 ligand compared to the average of the surrounding side chains should be presented. This should be done for all three liganded structures (B-factors of protein, solvent and ligands should be given separately in the Crystallographic Table).

2) The type of dimer referred to as an X shape is more technically referred to as a domain-swapped dimer where one domain makes close contacts to a second domain from a different chain

3) Page: 7- The authors state that “Sirohydrochlorin is the most tightly-bound, based on strong density and higher occupancy (Figure 2e).” It is not reasonable to make assessment of affinity based purely on occupancy and quality of density as the resolution of the X-ray crystal structures and competing molecules in the crystallization milieu have direct effect on both.

4) Page 7- The authors state “Co-sirohydrochlorin is the most stable of the molecules but is not bound deeply in the open active site cavity so the density is even more diffuse than that for precorrin-2, but sufficient to allow us to assign the orientation of the central plane of the tetrapyrrole as nearly orthogonal to the precorrin-2 and sirohydrochlorin binding poses (Figure 2e).” I think Figure 3f is

meant here and the density of Co-sirohydrochlorin shown in the figure looks stronger not more diffuse than that of precorrin-2. Again omit maps are essential here for the reader to assess occupancy and orientation.

5) Page: 10- “Both R260A and R261A CysG are overall similar to wild-type, with an RMSD of less than 1 Å after superimposition (Figure S2a).” As molecular replacement phasing is used then there should be some note that the rmsd value is biased somewhat by the use of the wild type structure in phasing

6) Page: 11- The authors state “Indeed, superimposition of an NADH-bound subunit shows that C15 is close to the nicotinamide ring that will abstract the hydride to make sirohydrochlorin (Figure 4a). Flattening of the ring upon conjugation moves C15 away from the NADH (Figure 4b).” Can the authors please provide the distances here rather than saying “close to” and “away from”? It would also be important to have the coordinate error reported for each of these structures so that differences can be assessed in context.

7) Page: 12- “In the open active site, the P133G variant has the same structure as the wild-type enzyme, suggesting that the interactions between the helical domain and the dimerization domain are important for preforming the closed active site. Can this be clarified- it is not clear how it follows from the previous statement that the in P113G the closed active site hydrophobic substrate binding platform is distorted.

8) Page: 13- "when a loop including D104- A105-P106-K107 (DAPK loop) flips over to lock the tetrapyrrole in the active site (Figure 3b)." The text should call out Fig 5b. Also the legend to Figure 5b does not describe that the two loops from apo and bound structure are shown and does not indicate the color scheme for them. The wording of the corresponding paragraph should be clarified- I take it Pro 106 acts as a steric block to prevent methylated tetrapyroles at C20 from binding. But the wording outward makes it seem like the distance is increasing? Please clarify,

9) The paragraph starting "One longstanding question about CysG’s function is how it binds to the metal ion for insertion and how the enzyme selects for iron over cobalt, given its ability to act as an iron and cobalt chelatase. D104 has been implicated in that activity" then goes on to describe the activity of D104 site directed mutants and their structures. Although the findings are consistent with the conformational changes described for the DAPK loop, it has nothing to do with the metal selectivity. The findings on these mutations are should be put in the appropriate context

Minor typos etc.
Throughout the manuscript proponyl group should be propionyl group (there is a carboxylate at the end).

Page: 5 "of the numerous amino acids that makeup the active site" should read "of the numerous amino acids that make up the active site"

Page: 6
“substrate/product that persisted after backsoaking" should read for clarity "substrate/product and this color persisted after backsoaking”

Page 7- “CysG is also a sirohydrochlorin cobaltochelatase, although selective for Fe^{2+}”. This statement needs a reference

Page 7- “Two more arginines derive from CysGA” please clarify as the previous sentence does not discuss Arg specifically, it is unclear why these two are discussed separately

Page: 9- The authors point out “water-mediated hydrogen bonds to the amide hydrogens on P1141,

A1281, G1301, and S132. The amide N on proline is not protonated in a peptide bond.

Page: 14- Uro’gen III methylation is catalyzed by a dimeric SUMT in all known pathways that is homologous to SUMTs involved in Vitamin B12 biosynthesis" should probably read "Uro’gen III methylation is catalyzed in all known pathways by a dimeric SUMT homologous to SUMTs involved in Vitamin B12 biosynthesis”

The legend for figure 4 does not describe the differential coloring of the structures overlayed.

Figure S1a legend needs correction" Sirohydrochlorin would interaction with S128 in the wild-type enzyme" should read “ would interact with”

In the crystallographic Table check sig figs- for instance for one structure resolution is given as 2.486 - 2.4

We would like to thank the reviewers for their thoughtful comments. We have responded to each, marked in blue.

Reviewer #1 (Remarks to the Author):

The manuscript concerns investigations into the mechanism of CysG, the siroheme synthase multifunctional enzyme that transforms uroporphyrinogen III into siroheme. The enzyme performs methylations at C2 and C7 of the macrocycle before catalysing dehydrogenation and chelation reactions. The latter two reactions are performed in the N-terminal region of the protein within a single site. The authors have undertaken a significant amount of work including crystallography, mutagenesis and enzymology in order to try and compose a molecular overture of events to explain how this enzyme is able to perform the dehydrogenation and chelation reactions. Excitingly, the authors have managed to generate substrate and product complexes of the enzyme and from this are able to provide new insights into the action of the enzyme. This works makes a significant advance to the area and represents a fairly complete piece of work. The work complements on-going studies elsewhere that aim to understand how cellular metal availability is related to enzyme activity so this paper is rather timely. There are some comments the authors may wish to address below:

1. Throughout text, replace typhimurium with enterica. Alternatively, for this serotype, Salmonella enterica subsp. enterica serotype Typhimurium, it can be abbreviated to Salmonella Typhimurium. Personally, I'd stick with S. enterica.

We have taken Dr. Warren's advice and replaced *typhimurium* with *enterica*.

2. In the abstract – it is stated "CysG's single active site … " but of course this is confusing. CysG has its methyltransferase active site, and is a dimer, so therefore technically there are 4 active sites??

Indeed, CysG's combined oligomeric state and gene-fusion product is complicated. We altered our description to clarify we are talking about the active site that is not a methyltransferase and replaced the term "single" with "common".

3. I am not familiar with proponyl as a term – propionyl is the more traditional spelling. Also, vitamin does not require a capital V. On trivial points, the term porphine (P11) refers to a metal-free porphyrin, rather than an isobacteriochlorin.

We have incorporated these changes throughout the manuscript

4. It is stated on Page 3 – that siroheme is more like vitamin B12 in terms of having propionyl and acetyl side chains. Given that B12 is ring contracted – a better comparison would be with F430.

We have incorporated this comparison.

5. The results are well aid out and nicely explained. Given that the precorrin-2 density is so weak,

I'm not sure that the authors can raw too much concerning its actual position and distortion – most of this is inferred. Similarly, I'm not sure they can, with real accuracy, say that the C15 position is located more closely located to the NAD than sirohydrochlorin, especially given that the NAD is modelled.

We agree that the density for precorrin-2 is weaker than for sirohydrochlorin. We screened over a dozen crystals in an attempt to obtain a structure with higher occupancy, so we are quite familiar with the solvent pattern in the cavity. This dataset was uniquely distinguishable from the typical pattern of waters/buffer molecules that bind in the cavity and was assessed by Phenix in calculating the newly-presented Polder omit maps as "likely to be the assigned molecule". Further, the changes we see to the overall protein fold in closing the helical domain around the tetrapyrrole, now shown in Figure S4, are also present in the precorrin-2 structure. To address the concern about the modelled NAD(H), we teamed up with Dr. Yu Chen from University of South Florida who is an expert in structure-based drug design. He and his student Michael Kemp computationally-docked NAD^+ against our experimentally-determined precorrin-2-bound structure and NADH against our experimentally-determined sirohydrochlorin structure. They used a computationally-docked sirohydrochlorin structure as a control to allow us a measure of the docking accuracy, given the complexity of the molecules in question. This new data is presented on page 12, in updated Figure 5 (old Figure 4), new supplemental Figure S5. Now, the discussion on page 16 focuses on the changes to the structure upon tetrapyrrole binding that impact NAD(H) binding and its relationship to the tetrapyrrole. Interestingly, these new observations explain the experimental result that the tetrapyrrole and NAD(H) molecules do not bind the same active site at the same time.

6. In the discussion, I'd point out that the CysG/Met8p chelatases represent a unique class III of chelatase, structurally distinct from the class I and II chelatases (see Brindley et al, 2003, J Biol Chem. 278, 22388-95).

This detail is now specified in the introduction and discussion.

7. I would try and compare the two aspartates (D104/262) found above the tetrapyrrole substrate in CysG to the residues found in CbiK/CbiX (two His; see Romão et al (2011), PNAS 108(1):97-102) and the ferrochelatases (His and Glu; Medlock et al 2007, PNAS 104(6):1789-93)

Indeed, the similarity struck us, as well, which is why we investigated the overall importance of D262 by mutagenesis, in particular given that it comes from the SUMT so is not present in Met8p. We originally reported that both the D262A and D262N variants were able to complement the CysG-deficiency, albeit with lower activity for both the dehydrogenation and chelation activities. We added data in the new Figure 4 showing that the D262N variant is more sensitive to cobalt dose than CysG, similar to the P133G variant. We included the comparison to the other chelatases in the results (page 14/15), where we also explain the results of the cobalt-challenge experiment.

While it is tempting to propose that this cobalt sensitivity arises from alteration of the secondary ligand, we felt this was too strong of a conclusion for a few reasons. 1) The D104N variant shows the same dose-response to cobalt as CysG, although it is impacted considerably by growth on M9 media without added cobalt. The preponderance of evidence supports that is that this is a true metal ligand, so if altering it to an asparagine does not affect cobalt sensitivity, then it is difficult to argue

that D262 would have that effect. 2) D262 is over 4 Å away from the water molecule that is mimicking the pre-inserted metal, too far in its present conformation to be making a hydrogen bond with the water, like D104 is. and 3) D262A retains activity, unlike D104A. Rather, we interpret the role of D262 as helping position an arginine to orient the tetrapyrrole in the active site. We included in the discussion further comparison with CbiX/K on page 16 because it also highlights other interesting observations about how sirohydrochlorin is oriented in the different chelatase active sites.

8. Finally, to put this back into a cellular context – I'd try and relate what you have found with the need to be able to bind Fe within the cell and relate this to the available intracellular metal concentration – see Osman et al (2019), Nat Chem Biol 15: 241-249.

We thank Dr. Warren for his suggestions in developing our discussion. Our added analysis of the amino acid variants that influence iron selectivity (new Figure 4) allows us to address this with additional experimentation, which is now included in the discussion on page 21.

Reviewer #2 (Remarks to the Author):

The manuscript by Pennington et al. describes a very thorough study of the enzyme SiroHeme and its interactions with the changing prosthetic group. There are at least eleven diffraction and refinement data tables (Table 1 type reporting) with resolutions better that 2 Å and good refinement statistics.

The amount and the quality of information is outstanding. Nevertheless, it is difficult to present this amount of information in a way that makes it easy for the reader. This reviewer thinks that reading pages of results on eleven structures (or more) is difficult. The manuscript would be highly improved if the format is changed to make the section after the Introduction a Results and Discussion. Combining these two sections early in the manuscript will allow the reader to understand the changes described in the present Results section as they are presented as opposed to learn about them and their implications only after all observed changes are described.

We thank Reviewer 2 for his/her comments. At the editor's recommendation, we will respectfully leave the manuscript with a separate Results and Discussion. We endeavored to streamline the manuscript and feel the additional biochemical analysis of the iron-selectivity mechanism puts the implications of our structural observations in perspective.

Reviewer #3 (Remarks to the Author):

The manuscript presents X-ray crystal structures of Salmonella typhimurium siroheme synthase (CysG) liganded to substrates product and metals. CysG has a bifunctional active site that catalyzes the final two steps of siroheme production, NAD+-dependent dehydrogenation and iron chelation. Currently, it is not known how a single active site performs these different chemistries. Semi-quantitative assay of complementation and of the two activities are presented to test the importance of active site residues using site-directed mutagenesis. The topic of the manuscript should be of interest to a broad readership on enzyme mechanisms, iron utilization, and metabolic pathways. Generally, the manuscript is well written and the assay work informative but a lack of information

for the X-ray crystallographic methodology makes it impossible to properly assess the technical merit. As detailed below, the authors should present debiased omit maps and information about the B-factors of the ligandsin order to confirm the presence of the ligands, which are the basis for the findings. Indeed, the electron density that is presented for the structure of the complex with precorrin-2 appears very poor and raises serious concerns about this particular liganded structure. The authors might consider the following points (page numbers from PDF for review):

Major concerns:
1) The electron density shown in figure 2d,e,f is from 2Fo-Fc maps To adequately assess the ligand density a debiased polder or simulated annealing omit maps or composite omit maps should be shown here or in supplementary materials (and it would be best if they were in stereo view). Indeed, the density for the structure with precorrin-2 is extremely disconnected and although the authors discuss this point, without additional maps and information on temperature factors there cannot be confidence in the interpretation. The additional maps are of great importance - on the precorrin-2 global structure validation metrics from the PDB validation report are below average for the resolution in all but one category. Omit maps and the average B-factor of the precorrin-2 ligand compared to the average of the surrounding side chains should be presented. This should be done for all three liganded structures (B-factors of protein, solvent and ligands should be given separately in the Crystallographic Table).

We have now specified B-factors for precorrin-2, sirohydrochlorin, *S*-adenosyl homocysteine (bound to $CysG^A$ but not discussed in this manuscript), and co-sirohydrochlorin, as well as solvent, in Table S2. Average B-factors for suppording tetrapyrrole ligands are specified in the figure legends. We re-generated Figure 2 using the suggested Polder omit maps and are submitting the coordinates as well as FEM $2F_o$-F_c (less biased than σ_a-weighted $2F_o$-F_c map for admittedly weak density, Afonine, *et al* Acta Cryst D 2015) and Polder omit maps for the reviewer's benefit (precorrin-2, sirohydrochlorin and co-sirohydrochlorin maps only – we are happy to submit this for all amino acid variants upon request). Structure factors for all structures have been deposited with the PDB. We have created three movies in which the view of the active site rocks to help the readers visualize the data.

2) The type of dimer referred to as an X shape is more technically referred to as a domain-swapped dimer where one domain makes close contacts to a second domain from a different chain

We updated this description as recommended.

3) Page: 7- The authors state that "Sirohydrochlorin is the most tightly-bound, based on strong density and higher occupancy (Figure 2e)." It is not reasonable to make assessment of affinity based purely on occupancy and quality of density as the resolution of the X-ray crystal structures and competing molecules in the crystallization milieu have direct effect on both.

We adjusted our description of the binding, as recommended.

4) Page 7- The authors state "Co-sirohydrochlorin is the most stable of the molecules but is not bound deeply in the open active site cavity so the density is even more diffuse than that for precorrin-2, but sufficient to allow us to assign the orientation of the central plane of the

tetrapyrrole as nearly orthogonal to the precorrin-2 and sirohydrochlorin binding poses (Figure 2e).” I think Figure 3f is meant here and the density of Co-sirohydrochlorin shown in the figure looks stronger not more diffuse than that of precorrin-2. Again omit maps are essential here for the reader to assess occupancy and orientation.

We replaced the maps with Polder omit maps and adjusted our description as recommended. The correct figure, Figure 2f, is now called.

5) Page: 10- “Both R260A and R261A CysG are overall similar to wild-type, with an RMSD of less than 1 Å after superimposition (Figure S2a).” As molecular replacement phasing is used then there should be some note that the rmsd value is biased somewhat by the use of the wild type structure in phasing

Yes, model bias is always a concern, which is why we refined all of our structures independently and using the best-practice methods of monitoring the R_{free} as well as R_{factor}. The main reason to include structures of the variants was to show that we could crystallize them and determine their structures, indicating the variant proteins were folded properly. That is, we wanted to verify that the measurements we made that showed reduced activities arose from altering the chemistry of the active site rather than destabilizing the protein fold. Further, we saw interpretable alterations in the structure of the P133 variants in the un-refined molecular replacement map (see Reviewer Figure 1, below), showing that the molecular replacement structure solution was sufficiently unbiased to reveal meaningful structural differences. That being said, to address the reviewer’s concern, we used Met8p as a reference in place of CysG and determined the RMSD between all of the $CysG^B$ variants and Met8p. Any gross structural deviation would result in a larger RMSD between the variants and Met8p than WT versus Met8p, given any subunit combination. None did – they were all within the standard deviation of the average RMSD. This information is now included in Supplemental Table S2.

Reviewer Figure 1: Maps calculated after molecular replacement of the P133G variant against the CysG structure but before refinement/sequence alteration revealed meaningful 2Fo-Fc (blue) and Fo-Fc (positive density is green, negative density is red) that could be interpreted as a substantive difference in the loop that contains amino acids 133. The un-modified search model is green, the modified/refined final model is yellow.

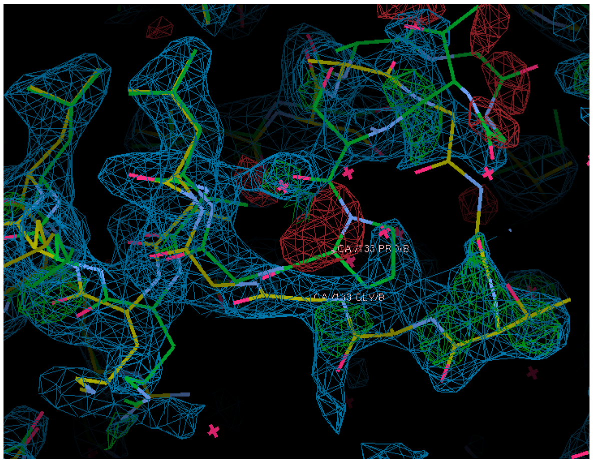

6) Page: 11- The authors state “Indeed, superimposition of an NADH-bound subunit shows that C15 is close to the nicotinamide ring that will abstract the hydride to make sirohydrochlorin (Figure 4a). Flattening of the ring upon conjugation moves C15 away from the NADH (Figure 4b).” Can the authors please provide the distances here rather than saying “close to” and “away from”? It would also be important to have the coordinate error reported for each of these structures so that differences can be assessed in context.

We have substantially altered this part of the manuscript in light of the new computational analysis (page 12 and 16). Given reviewer 1’s comment 5, we have respectfully declined to offer distances in order that we not over-interpret our model. Nevertheless, we endeavored to make our presentation of the results and discussion more specific to describe the structures.

7) Page: 12- “In the open active site, the P133G variant has the same structure as the wild-type enzyme, suggesting that the interactions between the helical domain and the dimerization domain are important for preforming the closed active site. Can this be clarified- it is not clear how it follows from the previous statement that the in P113G the closed active site hydrophobic substrate binding platform is distorted.

We added an additional supplemental Figure S3 to show how the platform is unwound in one of the active sites (corresponding to the same closed active site where precorrin-2 and sirohydrochlorin bind) but not in the open active site. We clarified our interpretation of this observation in light of the new Co^{2+} competition data, see page 18.

8) Page: 13- "when a loop including D104- A105-P106-K107 (DAPK loop) flips over to lock the tetrapyrrole in the active site (Figure 3b)." The text should call out Fig 5b. Also the legend to Figure 5b does not describe that the two loops from apo and bound structure are shown and does not indicate the color scheme for them. The wording of the corresponding paragraph should be clarified- I take it Pro 106 acts as a steric block to prevent methylated tetrapyroles at C20 from binding. But the wording outward makes it seem like the distance is increasing? Please clarify,

We have corrected the figure citation (now Figure 6b) and clarified the legend. We also clarified our description of the motion by making it clear that P106 must move out from its apo position to make space for the very large, flat sirohydrochlorin, see page 13.

9) The paragraph starting "One longstanding question about CysG’s function is how it binds to the metal ion for insertion and how the enzyme selects for iron over cobalt, given its ability to act as an iron and cobalt chelatase. D104 has been implicated in that activity" then goes on to describe the activity of D104 site directed mutants and their structures. Although the findings are consistent with the conformational changes described for the DAPK loop, it has nothing to do with the metal selectivity. The findings on these mutations are should be put in the appropriate context

We have added a new set of experiments to address metal ion specificity, allowing us to address these questions specifically so this section has been substantially revised. In these experiments, we

challenged the amino acid variants that retained the ability to complement a *cysG*-deficient strain of *E. coli* to grow under increasing concentrations of Co^{2+} (Figure 4). CysG-complemented *E. coli* show only a moderate effect at even 100 μM Co^{2+}, still selecting for Fe^{2+} that either comes from intracellular pools or scavenging from the reagents used to make the minimal media. Similarly, although R261A has a 20-fold reduced chelatase activity, it responds to increasing Co^{2+} dose in a similar fashion. D104N is significantly impacted by growth on minimal media but responds to the Co^{2+} dose in a similar way to CysG and R261A. Point variants at positions that impact the binding of sirohydrochlorin, either in forming the platform (P133G/H) or interacting with the carboxylates (R260A/D262N, albeit indirectly), are all more dramatically impacted by increasing Co^{2+}. We now discuss the implications of the observations on pages 19-20.

Minor typos etc.
Throughout the manuscript proponyl group should be propionyl group (there is a carboxylate at the end).

Fixed.

Page: 5 "of the numerous amino acids that makeup the active site" should read "of the numerous amino acids that make up the active site"

Fixed.

Page: 6 “substrate/product that persisted after backsoaking" should read for clarity "substrate/product and this color persisted after backsoaking”

Thank you for the suggestion to clarify our language. It is now implemented.

Page 7- “CysG is also a sirohydrochlorin cobalt chelatase, although selective for Fe2+”. This statement needs a reference.

We added references for both assertions, first, that it is a cobalt chelatase: 16) Fazzio, T. G. & Roth, J. R. Evidence that the CysG protein catalyzes the first reaction specific to B12 synthesis in Salmonella typhimurium, insertion of cobalt. *J Bacteriol* 178, 6952-6959 (1996); second, that it is selective for iron 11) Warren, M. J. *et al.* Gene dissection demonstrates that the Escherichia coli cysG gene encodes a multifunctional protein. *Biochem J* 302 (Pt 3), 837-844 (1994) and 17) Woodcock, S. C. *et al.* Effect of mutations in the transmethylase and dehydrogenase/chelatase domains of sirohaem synthase (CysG) on sirohaem and cobalamin biosynthesis. *Biochem J* 330 (Pt 1), 121-129 (1998).

Page 7- “Two more arginines derive from CysGA” please clarify as the previous sentence does not discuss Arg specifically, it is unclear why these two are discussed separately

Most of the charged groups that interact with the carboxylates are lysines or arginines, as shown in Figure 3. To clarify, we removed the word “more” from that sentence to specifically highlight those arginines, which stood out to us because they derive from the $CysG^{A}$ (*ie* the methyltransferase module), which is interesting because this is not part of Met8p. We discuss the

implications of this in terms of the evolutionary benefit of the gene duplication-fusion that resulted in CysG.

Page: 9- The authors point out “water-mediated hydrogen bonds to the amide hydrogens on P1141, A1281, G1301, and S132. The amide N on proline is not protonated in a peptide bond.

We removed P114 from the list.

Page: 14- Uro’gen III methylation is catalyzed by a dimeric SUMT in all known pathways that is homologous to SUMTs involved in Vitamin B12 biosynthesis" should probably read "Uro’gen III methylation is catalyzed in all known pathways by a dimeric SUMT homologous to SUMTs involved in Vitamin B12 biosynthesis”

Thank you again for your help in clarifying our descriptions. The change is made.

The legend for figure 4 does not describe the differential coloring of the structures overlayed.

I have added this to the figure legend.

Figure S1a legend needs correction" Sirohydrochlorin would interaction with S128 in the wild-type enzyme" should read “would interact with”

Correction made.

In the crystallographic Table check sig figs- for instance for one structure resolution is given as 2.486 - 2.4

We went through Table S2 and endeavored to adjust the output from Phenix to more appropriate significant figures.

Reviewers' comments:

Reviewer #1 (Remarks to the Author):

The authors appear to have addressed all the issues that were raised and have produced a significantly improved manuscript.

Reviewer #3 (Remarks to the Author):

The revised manuscript by Pennington et al. has addressed some, but not all of this reviewer's concerns. There is still concern about the density for the precorrin-2 and Co-sirohydrochlorin, as the contour level of the Polder maps is lower than usually displayed. Also, in reading the comparison of the B-factors to that of protein (Fig 2 legend) one would assume that the occupancies were set as 1.0, (see comment below) then the B-factors for the density are reasonable. However, looking at the PDB file for Co-sirohydrochlorin it seems that the occupancy was set to 0.5- so the B-factors would be even higher with an occupancy of 1.0. The authors are cautious about overinterpretation in their wording, but still I feel that they should be forthcoming about occupancy and B-factors resulting from the poor density. It is difficult to judge even with validation reports, how good the data for the ligands themselves is- as the majority of the total diffraction comes from the protein. The authors should also check throughout to remove colloquial phrasing as discussed below. Comments the authors should consider follow (page numbers from pdf merged for review):

Page: 4- The authors replaced description of dimer in results as domain swapped- but in the introduction here the molecule is still described as "an X-shaped molecule, where positioning of the Rossmann fold from one subunit over the helical domain from the opposing subunit creates a large cavity. I think it should also be described here as a domain-swapped dimer.

Page: 18- "the activity site less rigid by unfolding helix 6" should probably read "...active site..."

Please check throughout typos
Page 19- Please remove imprecise language- "The bulkier histidine projects into the active site, landing about where the pyrrole nitrogens are when the molecule binds, so would also disrupt tetrapyrrole binding and release" should remove the word landing and replace with positioned and end of sentence should be corrected. Should read something like- "The bulkier histidine projects into the active site, and is positioned in place of the pyrrole nitrogens when tetrapyrole binds, thus likely disrupting tetrapyrrole binding and release."

Page: 38- The polder maps in Figure 2 d-f are all contoured at 2 sigma- but it is more usual to contour at 3 sigma- Is the quality of the density good at three sigma as well? If so, please display contoured at 3 sigma. Also please note in the Figure legend of Figure 2 when B-factors are described that the occupancy of the ligands was set to 0.5. It may be more appropriate to set the occupancy to 1.0 and report the higher B-factors. The authors should be forthcoming about the quality of the ligand density.

Page: 39- Figure 3B- proline 133 is drawn as protonated- please remove the proton

Again, we thank reviewer 3 for the thorough reading of our revised manuscript. All changes to the text are marked in blue, as are our responses below.

The revised manuscript by Pennington et al. has addressed some, but not all of this reviewer's concerns. There is still concern about the density for the precorrin-2 and Co-sirohydrochlorin, as the contour level of the Polder maps is lower than usually displayed. Also, in reading the comparison of the B-factors to that of protein (Fig 2 legend) one would assume that the occupancies were set as 1.0, (see comment below) then the B-factors for the density are reasonable. However, looking at the PDB file for Co-sirohydrochlorin it seems that the occupancy was set to 0.5- so the B-factors would be even higher with an occupancy of 1.0. The authors are cautious about overinterpretation in their wording, but still I feel that they should be forthcoming about occupancy and B-factors resulting from the poor density. It is difficult to judge even with validation reports, how good the data for the ligands themselves is- as the majority of the total diffraction comes from the protein. The authors should also check throughout to remove colloquial phrasing as discussed below. Comments the authors should consider follow (page numbers from pdf merged for review):

Page: 4- The authors replaced description of dimer in results as domain swapped- but in the introduction here the molecule is still described as "an X-shaped molecule, where positioning of the Rossmann fold from one subunit over the helical domain from the opposing subunit creates a large cavity. I think it should also be described here as a domain-swapped dimer.
We have made this additional change on page 4.

Page: 18- "the activity site less rigid by unfolding helix 6" should probably read "...active site..."
Thank you again for your careful reading – this change has been made.

Please check throughout typos.
We asked our departmental editor to read through for typos.

Page 19- Please remove imprecise language- "The bulkier histidine projects into the active site, landing about where the pyrrole nitrogens are when the molecule binds, so would also disrupt tetrapyrrole binding and release" should remove the word landing and replace with positioned and end of sentence should be corrected. Should read something like- "The bulkier histidine projects into the active site, and is positioned in place of the pyrrole nitrogens when tetrapyrole binds, thus likely disrupting tetrapyrrole binding and release."
We made this suggested change.

Page: 38- The polder maps in Figure 2 d-f are all contoured at 2 sigma- but it is more usual to contour at 3 sigma- Is the quality of the density good at three sigma as well? If so, please display contoured at 3 sigma. Also please note in the Figure legend of Figure 2 when B-factors are described that the occupancy of the ligands was set to 0.5. It may be more appropriate to set the occupancy to 1.0 and report the higher B-factors. The authors should be forthcoming about the quality of the ligand density.
We added information about the occupancies and a second map for each at 3s threshold.

Page: 39- Figure 3B- proline 133 is drawn as protonated- please remove the proton
We made this change.
